# Customer's Channel Selection Behavior on Purchasing Standardized and Customized Products: Optimized Prices and Channel Performances

**DOI:** 10.3389/fpsyg.2022.871322

**Published:** 2022-10-11

**Authors:** Bisheng Du, Zhenfang Li, Jia Yuan, Jingyi Zheng, Wenwen Shu, Yao Jin

**Affiliations:** ^1^Business School, Ningbo University, Ningbo, China; ^2^Center for Collaborative Innovation on Port Trading Cooperation and Development, Ningbo University, Ningbo, China; ^3^Stockholm Business School, Stockholm University, Stockholm, Sweden; ^4^School of Civil Engineering and Environment, Ningbo University, Ningbo, China; ^5^Law School, Ningbo University, Ningbo, China

**Keywords:** customer behavior, channel selection, standardized product, customized product, dual channel

## Abstract

Nowadays, the traditional production is unable to meet the new diverse needs of target customers. In the current customization era, more and more companies are required by customers to provide more desirable customized products. However, research on customization and standardization based on quantitative analysis has drawn little attention in the literature of dual channel supply chain. In this paper, we study the effect of adopting a dual channel supply chain on the performance of a two-level system (manufacturer-retailer) by using a novelty quantitative approach. We try to analyze the system to get optimal prices and maximize profits, where manufactures offer both standardized and customized products via their traditional and customized channels, respectively. We build a Stackelberg game mode to construct a centralized and a decentralized dual channel scenarios. Furthermore, we study the effects of the different channel structures on price, degree of customization, degree of standardization, and supply chain profitability. We also analyze the effects of both standardized and customized demand sensitivities on their prices and profits. Eventually, we introduce a cost-sharing coordinating contract to optimize the channel's performance. We find that the potential market demand for customization affects the price of customized products and the profits of customized channels. Compared with the decentralized dual channel case, the cost-sharing contract can achieve higher total channel profits. In the cost-coordination case, there is an optimal range for the proportion of standardized costs borne by manufacturers.

## 1. Introduction

In recent years, with the rapid development of internet technology and many advanced technologies, consumers' desire for customized products has received unprecedented attention. Many companies are willing to understand the real needs of consumers and provide them with excellent service experience of customized products. On the other hand, consumers are also prefer to pay the corresponding premium for customized products. What's even more incredible is that, consumers are looking forward to actively participate in the product design and production process. The result of this is, the manufacturing process of products is increasingly directly driven by the individual needs of consumers.

Meanwhile, advances in manufacturing and information technology make it possible to efficiently produce customized product. Throughout the history of production, the Industrial production modes have evolved from the craft production mode to the mass production mode during the Industrial Revolution, then developed to the mass customization mode, and eventually to the customized production mode. In the customized production mode, companies will pay more attention to collect consumers' preferences and analyze their potential needs. They would like to fit consumers' demand with their customer-oriented, multi-variety, small batch production mode. The transformation of production mode is not only the production strategy of some companies, but the industrialization strategy of many countries. Many countries have published their re-industrialization strategies in the past years. For instance, Germany published the Industry 4.0 strategy in 2013 while China announced the Made-in-China 2025 (a 10-year national plan) in 2015, see Li (2018). Meanwhile, the United States, United Kingdom, France and other developed countries also have their re-industrialization strategies. The Made-in-China 2025 plan lays down China's approach to move up on the value chain and transform itself from a world manufacturing production workshop into a world-class industrialized giant. It focuses on binding the new generation of information technology, such as big data and cloud computing, with the modern manufacturing industry, and making continuous transformations and upgrades.

Product customization is increasingly becoming an important feature of the coordinated development of manufacturing industry and information technology. It's obvious to see that information technology is a powerful tool for the development of customer service. It does not only reconstruct the design process and improve the efficiency of production, but also push the digital and customized development of product design. In the industries of clothing, furniture, household appliances, automobiles, among others, design-based customization on information technology becomes an essential method for companies to achieve transformation. For instance, we can design a customized watch with our favorite frames and dial color, we can choose the straps with metal or leather under different colors at the website timissimo.com. Besides, customized slogans can even be engraved on the back of the watch. Therefore, customization is a multi-variety, small-batch-production mode which follows the personalized needs of customers, and reflects the people-oriented business philosophy of the companies. Additionally, it is an important manifestation of the core competitiveness of companies under the Industry 4.0 scheme. There are many advantages in customization. First, to improve the customer loyalty. Customers can make good use of the convenient information channel to timely communicate with product providers or design departments before purchase (greatly reducing the time to select products). Moreover, customers can also examine the production process through the network system. In the production process, any problem can be solved directly. Therefore, the customized business mode could improve customer satisfactions with their finished products. Then the customers' loyalty to the company will be greatly enhanced. Uniqlo's UT custom area allows customers to create their own designs, print and embroider. In addition, uniqlo relies on LBS positioning service to enable users to find nearby stores and to place orders online and pick up goods in stores, which makes the relationship between consumers and company closer. However, in the traditional channel, consumers can only buy high-rating clothes, and the company will not provide other services. Second, to reduce the inventory cost. In the past, companies have to produced products and sold them later, and the products may even remain unsold. The unsold products generate inventory cost heavily. With the development of information technology, companies can allocate their production lines according to their customer needs. Therefore, it can reduce their inventory, improve their inventory turnover, and greatly improve the efficiency of capital use. Third, to reduce the cost of sales. Customization enables manufacturers to communicate with their end-consumers directly, which eliminating the cost of intermediary distributors. In addition, the goods are produced according to the customers demand or design. If the quality is guaranteed and the price is reasonable, these products will reach the customers' expectations naturally. Thus, customization can basically avoid the cost of sales and promotion. Companies can introduce customization platforms and design various customer interaction tasks. Accordingly, the customers can participate in the products' customization process. West Lake No.1, a customized silk scarf design and retail platform for consumers based on artificial intelligence technology, has been officially launched in retail stores in Hangzhou. This platform can realize the real-time communication between AI designers and consumers, and conduct one-to-one customized design and production of silk scarves according to consumers' own characteristics, which greatly reduces the operating cost of manufacturing and retail enterprises.

The innovation mode of customization driven by customers is derived from the new era characters. There are more openness, interconnections, sharing, and experience-oriented. It is a reverse-customization production mode, known as C2B (Customer to Business) mode, that subverting B2C (Business to Customer) mode. The concept of C2B originally started in the e-commerce area, which means that the customers gather together and bargain with the sellers collectively to transfer price advantage from the manufacturers to themselves, see Thirumalai and Senthilkumar (2017). The C2B mode connect with the mass customization, which is defined as C2B2M-MC (Customer to Business to Manufactory-Mass Customization) mode. Customer drive is not only reflected in the purchase, sale of goods, operation and management, but also in the manufacturing process of products, see Salvador et al. (2009). Mass customization is a basic mode of production that generates highly customized products with the costs close to the mass production ones, see Kotha and Pine (1994). The essence of mass customization is to discover the personalized demand of customers and provide targeted solutions. Mass customization is not a complete customization, but a limited one based on the selection of a number of products and services. Furthermore, C2B is connected with mass personalization, which is defined as C2B2M-MP (Customer to Business to Manufactory-Mass Personalization) mode. The original customized mode was dominated by companies, with customers choosing only a limited mix of existing product modules. Therefore, C2B should extend from the mass customization to the mass personalization, that is, the transition from product modular customization (MC) to product fully customization (MP), see Zhang et al. (2019). Product differentiation focuses on the individual customer, rather than the whole market segments. C2B2M-MP can coordinate the needs of a single customer and produce customized products within the capacity of the factory to meet the consumers' needs. C2B developed into a C2M (Customer to Manufactory) mode. C2M is also called short circuiting economy, see Skold (2010). C2M directly connects factories and customers, that eliminates the intermediate circulation of products, realizes the zero inventory of customer orders, and meets the personalized needs of customers. Red Collar Group is an early adopter of this C2M customized business mode. Red collar is a large-scale garment manufacturer producing fine suits and other products. In the process of integration of industrialization and information, Red Collar built a clothing database system with independent intellectual property rights. It has developed a set of customization system based on the collected information and big data. Red Collar has been taking orders for customized clothes since 2003. There are more than 100 trillion pieces of data in its big database system. Customer customized requirements are submitted through a C2M platform, and the system automatically generates their orders instantaneously. This method of production breaks through the bottleneck of manual production. In this mode, customers participate in almost all of their processes such as design, manufacturing, logistics, sales and others, see Jia et al. (2016).

In practice, manufacturers in a variety of industries have developed their customized channels, while keep their distributor relationships and retail channels intact. Companies such as Dell, IBM, Nike, Hewlett-Packard, Apple, and Pioneer Electronics demonstrate the use of the dual channel mode, see Tsay and Agrawal (2009). While Nike maintains its traditional retail model, customers can purchase customized products at www.nike.com. These business transformation practices motivate us to generate the following research questions.

1. How do different channel structures affect the pricing mechanism, customization decisions, channel selection, and the overall supply chain performance?
2. What are the effects of different degree of customization and standardization on the dual channels?
3. How to coordinate the dual channel supply chains of standardized and customized products?

To address these questions, we considers three typical channel structures, the centralized dual channel (Scenario *C*) case, the decentralized dual channel (Scenario *E*) case and the cost-sharing dual channel (Scenario *O*) case. We investigate the impacts of channel structures on price, customized decision, and supply chain performance. Moreover, we demonstrate that the increasing speed of the decentralized scenario is greater than that of the centralized one. Because the decentralized channel price is more affected by both standardized and customized elasticity of demand. Comparing the overall supply chain performance under the centralized case and the decentralized case, our work shows that the overall supply chain performance can be increased with the introduction of the customized channel as well as the degree of customization. In practice, channel managers should strive to introduce and increase R&D investment in the customized channel and improve the level of product customization.

Additionally, we consider the impact of inconsistencies between the standardized elasticity of demand and the customized elasticity of demand on the supply chain decision making. Our results show that, in the centralized case, with the increase of the customized elasticity of demand, the prices of customized products keep rising, while the prices of standardized products remain relatively stable. Demand for the customized products grows faster than that for the standardized one. Then the overall supply chain performance increases. However, in the decentralized case, the channel profit does not increase significantly with the increase of the degree of customization. This result occurs because of the channel conflict between the customized channel and the standardized channel.

In the cost-sharing contract, the sales price is lower than that in the centralized case. The selling price under the coordination case is close to that under the decentralized one. Additionally, compared with the overall profit under the decentralized case, the overall profit under the cost-sharing contract achieves better. We consider that in the coordinated case, the manufacturer helps the retailer by covering part of the cost in the standardized channel. Therefore, the retailers have more incentives to invest in the construction of the standardized channel. When manufacturers absorbs the standardization costs of retailers within a moderate range, the total profit of the system is stable. When the value of the cost is too high, then the total profit of the system declines exponentially. Therefore, manufacturers can induce retailers to try harder to sell standardized products by setting a reasonable cost-sharing ratio theme.

The remainder of the paper is organized as follows. In section 2, we review the related literature. In section 3, we develop our mode including demand and profit functions, and the related assumptions. We also obtain the optimal and equilibrium outcomes of the centralized and decentralized supply chains, respectively. Moreover, we describe the impact of channel structure, the degree of standardization, the degree of customization, the customized elasticity of demand, and the standardized elasticity of demand on the supply chain decision making. In section 4, we analyze the mechanism of the cost-sharing contract to coordinate these two channels. Concluding remarks are presented in section 5.

## 2. Related Literature

Customized channels can create more profits for the manufacturers. However, the existence of customized channels affects the interests of retailers, which may lead to retailers' resistance to the customized channel. Such effect has a negative impact on the manufacturers' total profits. As a result, how to effectively coordinate the customized channel and the standardized channel is an important issue in the field of customized supply chain management. Many scholars have already analyzed this issue. At present, researches on customized supply chain mainly focus on,

1. Customization research from the perspective of consumers.
2. Pricing mechanisms of product substitution effect.
3. Conflict coordination mechanisms of dual channel supply chain.

In terms of customized research from the consumer's perspective, Bardakci and Whitelock (2004) discussed the attitudes of British consumers toward customized products and the influencing factors of mass customization, pointing out that the biggest influencing factor of customized products for consumers was the rise in prices. Yan et al. (2021) reviewed the literatures of additive manufacturing and 3D printing to reveal the state-of-art technologies on the production of customized products. Kumar and Ruan (2006) showed that the degree of brand loyalty and channel loyalty may influence the wholesale price, the retail price and the manufacturer's decision of introducing customized channels. Kurniawan et al. (2006) analyzed consumer decisions in product selection and customization tasks and found that consumers who participated in product customization were more satisfied with the product itself and the customization process. Li et al. (2020) study the perceived value and product involvement for customers to purchase customized garments in the fashion textile and apparel industry. Franke and Schreier (2008) found that customized products could increase consumers' willingness to consume among them. They found that the uniqueness of customized products played a major role and it could also affect consumers' positive experience and willingness to participate in product customization.

With regard to the research on pricing mechanisms for product substitution effects, Kuyumcu and Popescu (2006) studied the inventory management for the certainty of price of alternative products. The research showed that the problem of deterministic joint price inventory control with alternative products could be reduced to a pure pricing problem under the standardized regularization assumption of demand. If demand was uncertain and/or the product showed complementary effects, demand rationing could be profitable. Karakul et al. (2008) studied joint pricing and purchase volume modes for new and existing products with product alternatives. Liu et al. (2012) found that mass customization (MC) was a targeted industry practice. MC products returns were generally prohibited, therefore, MC retailers could gain a significant competitive advantage by providing consumer return policies. Through the establishment of a demand and revenue uncertainty analysis mode, they studied the optimal mechanism under the mean square error formula pricing, consumer returns and modularization three dimensions. Gupta et al. (2020) built a supply chain system with two suppliers and one retailer. Using the settings of Nash and Stackelberg games, they analyzed the impact of disruptions in supply capacity on pricing decisions for alternative products. Chen et al. (2013) consideres the pricing policy of a manufacturer in the supply chain. The manufacturer sold products to an independent retailer and also directly to consumers through Internet channels. In addition to the manufacturer's products, the retailer sold alternative products made by another manufacturer. They derived the existence and uniqueness conditions of the corresponding equilibrium solution for the Nash and Stackelberg games. Xiao et al. (2014) uses a Stackelberg pricing mode to investigate channel structure and in which the retail channel sold standardized products and the online channel offered customized products. They found that the unit wholesale and the retail prices of a standardized product sold through a retail channel were increased due to the addition of the direct channel for customized product. Savaskan and Van Wassenhove (2006) studied the problems of joint pricing and product technology selection faced by manufacturers when introducing remanufactured products into differentiated product markets.

Eventually we review the literature on dual channel supply chain conflict coordination. Boyaci (2005) analyzed the dual channel conflict based on Nash game, and explored the channel inefficiencies induced by the presence of simultaneous vertical competition (double-marginalization) and horizontal competition (substitutability). Boyaci suggested that combined contracts could better coordinate dual channel systems. Tsay and Agrawal (2009) argued that both online direct channels and traditional distribution channel had externalities in promotion, but retail channel promotion had a cost advantage. They proposed that the combination of the buyback price and the total wholesale price contracts could coordinate the supply chain. Chiang (2010) also designed a combination contract of inventory cost and network channel revenue sharing to solve the coordination problem of the dual channel. He further verified that the combined contract could enable the coordinated operation of the network channel and the traditional channel. Kurata et al. (2007) studied dual channel operation under different pricing mechanisms and made comparative analysis with numerical simulation. He pointed out that a single wholesale price mechanism could not coordinate it, but the combination of wholesale price mechanism with price reduction or price increase compensation could effectively coordinate the dual channel system. Li et al. (2015) found that the price of standardized products offered by retailers did not necessarily fall due to the online offering of customized products. He also found that under certain conditions, both manufacturers and retailers saw increased profits when manufacturers offered customized products online.

The findings above provide a strong foundation for our research. Bardakci and Whitelock (2004), Kumar and Ruan (2006), Kurniawan et al. (2006), and Franke and Schreier (2008) mainly study the customization effects from the perspective of consumers. In this paper, we also discuss these effects when presenting our results. Moreover, our focus is not only on the relationship between consumer demand and products' prices, but also on the influence of different degrees of customization and standardization on demand and price. Kuyumcu and Popescu (2006), Savaskan and Van Wassenhove (2006), Karakul et al. (2008), Liu et al. (2012), Chen et al. (2013), and Gupta et al. (2020) mainly study the pricing mechanisms related to product substitution effects. Our work differs from this research stream in several respects. First, we do not only employ an inverse demand function to analyze the quantity decisions in a context of dual channel supply chains. Instead, we introduce the customized channel and the standardized channel scenarios. We analyzed the influence of different degree of customization and degree of standardization on the overall channel performance and obtain a more comprehensive outcome. Finally, we also consider both customized and standardized elasticity of demand. Boyaci (2005), Tsay and Agrawal (2009), Chiang (2010), and Kurata et al. (2007) study dual channel supply chain conflict coordination mechanisms. However, the relationship between the price, the degree of customization and the degree of standardization are not considered. Nevertheless, quite a number of researchers have focused on the study of dual channel coordination from the perspective of homogeneous products, but seldom make quantitative analysis on the internal mechanism of dual channel conflict. On this basis, less research is carried out on dual channel conflict coordination mechanisms based on customization and standardization. In the context of competition between the customized channel and the standardized channel, we establish a decision modes of price, degree of customization and degree of standardization. First, we analyze the impact of different channel structures on pricing, degree of customization, degree of standardization and supply chain profitability. Then we analyze the influence of different degree of customization and standardization on the optimal results. Finally, we design a cost-sharing contract to solve the imbalance problem between customized and standardized products.

## 3. Problem Formulation

We consider a supply chain system with customized and standardized channels, that consisting of two independent entities, a manufacturer and a retailer. To meet customers' demand and their preferences, the manufacturer adopts a dual channel strategy in which the manufacturer has a regular retailing channel and an online channel. The standardized products are sold by the retailer while the customized products are sold by the manufacturer. The manufacturer pays for the customized product cost and the retailer pays for the standardized product cost. We discuss the centralized case (Scenario *C*), the decentralized case (Scenario *E*), and the coordinated case (Scenario *O*) with the manufacturer as the leader and the retailer as the follower. Then we study the behavior of this system, analyze the optimal values of the decision variables under different conditions and measure the performance by using the total profit of the supply chain. Further, we examine the effects of different pricing and coordination mechanisms. *m* represents the degree of customization. From standardized products to modular customization to full customization, the larger *m* is, the higher degree of customization is. *e* represents the degree of standardization. The higher the degree of standardization, the clearer the product classification is and the more selective customers can be. We use the terms *D*_*e*_, *D*_*m*_ to indicate the demand of the standardized product and the demand of the customized products, respectively. The sale price of customized products is denoted as *p*_*m*_ and the sale price of standardized products is represented by *p*_*e*_. θ (0 ≤ θ < 1) denotes channel substitutability. The channels are demand interdependent (unless θ = 0), although α_*e*_ and α_*m*_ appeal to different market segments. α_*i*_ > 0 (*i* = *m, e*) represents the base demand of customized product and standardized product, respectively. λ_*i*_ (*i* = *m, e*) denotes the effect of an increase in the product's customization and standardization on each channel's demand. In Table 1 we listed all the necessary notations.

**Table 1:** Summary of notations.

**Notation**	**Description**
*U*	The utility for representative consumer
*p*_*e*_	Price of the standardized product
*p*_*m*_	Price of the customized product
*e*	Degree of standardization
*m*	Degree of customization
*w*	Wholesale price, *w* ≤ *p*_*e*_ *w* ≤ *p*_*m*_
*D*_*e*_	Demand for standardized products
*D*_*m*_	Demand for standardized products
α_*e*_	Base demand of the standardized product
α_*m*_	Base demand of the customized product
θ	Channel substitutability, 0 ≤ θ < 1
τ	Percentage of standardization costs borne by the manufacturer, 0 ≤ τ < 1
λ_*e*_	Standardized elasticity of demand
λ_*m*_	Customized elasticity of demand
*K*_*e*_	Unit standardized product investment
*K*_*m*_	Unit customized product investment

We adopt the framework established by Ingene and Parry (2004), which has been applied extensively in the field of operations and marketing management, see Cai (2010), Cai et al. (2015), Chen et al. (2017), and Snyder and Shen (2019), and we employ a similar utility function for the representative consumer as follows.


(1) $$\begin{array}{l} U\left(D_{m},D_{e}\right)=\alpha_{m}D_{m}+\alpha_{e}D_{e}-p_{m}D_{m}-p_{e}D_{e}-\frac{1}{2}D_{m}^{2}-\frac{1}{2}D_{e}^{2}\\ \;-\theta D_{m}D_{e}\\ \;+\lambda_{m}mD_{m}+\lambda_{e}eD_{e} \end{array}$$

Equation (1) implies that the representative consumer utility function is linearly dependent on price, degree of standardization and degree of customization. It also indicates that utility decreases in price while increases in the degree of standardization and the degree of customization. Moreover, the utility decreases in channel substitution θ and increases in the customized elasticity of demand λ_*i*_.

Therefore, we estimate consumers' demand functions by maximizing *U* with respect to *D*_*m*_ and *D*_*e*_, respectively.


(2) $$\begin{array}{l} \{\begin{array}{l} \frac{\partial U}{\partial D_{m}}=\alpha_{m}-p_{m}-D_{m}-\theta D_{e}+\lambda_{m}m=0\\ \frac{\partial U}{\partial D_{e}}=\alpha_{e}-p_{e}-D_{e}-\theta D_{m}+\lambda_{e}e=0 \end{array} \end{array}$$

In the following, the demand of the standardized channel is


(3) $$\begin{array}{l} D_{e}=\frac{1}{1-\theta^{2}}\left[\left(\alpha_{e}-\theta \alpha_{m}\right)-\left(p_{e}-\theta p_{m}\right)+\left(\lambda_{e}e-\theta \lambda_{m}m\right)\right] \end{array}$$

and the demand of the customized channel is


(4) $$\begin{array}{l} D_{m}=\frac{1}{1-\theta^{2}}\left[\left(\alpha_{m}-\theta \alpha_{e}\right)-\left(p_{m}-\theta p_{e}\right)+\left(\lambda_{m}m-\theta \lambda_{e}e\right)\right] \end{array}$$

We find that the requirements function for standardized and customized products is linear, as in Zhang et al. (2015) and Chen et al. (2017).

With the above demand functions, we formulate the individual profits in the supply chain. The manufacture's profit function is


(5) $$\begin{array}{l} \Pi_{M}\left(p_{m},m\right)=\left(w-c_{e}\right)D_{e}+\left(p_{m}-c_{m}\right)D_{m}-\frac{K_{m}}{2}m^{2} \end{array}$$

where the first term is the manufacturer's revenue of the standardized channels, the second term is the manufacturer's revenue of customized channels, and the third term is the manufacturer's customized cost. The retailer's profit function is


(6) $$\begin{array}{l} \Pi_{R}\left(p_{e},e\right)=\left(p_{e}-w\right)D_{e}-\frac{K_{e}}{2}e^{2} \end{array}$$

where the first term is the retailer's revenue of the standardized channels and the second term is the retailer's standardized cost. In Equations (5) and (6), *w* is the wholesale price which has been given. We define *c*_*m*_ as the unit cost of customized channel and *c*_*e*_ as the unit cost of the standardized channel. We mode costs of the customization and standardization as a quadratic function, which explains why increasing the degree of customization or the degree of standardization at a high level increases costs, and reduces returns, see Tsay and Agrawal (2009). The total profit of the dual channel supply chain π_*T*_ is given by the sum of Equations (5) and (6), as follows


(7) $$\begin{array}{l} \Pi_{T}=\Pi_{R}+\Pi_{M} \end{array}$$

We have the following assumes,

**Assumption 1**. All of the channel members are risk-neutral and completely rational. In other words, the manufacturer and the retailer make choices to pursue their maximize expected profits.

**Assumption 2**. The products selling through standardized channel and customized channel are different. The manufacturer determines the wholesale price per unit *w* and the price of customized products *p*_*m*_, while the retailer determines the channel retail price *p*_*e*_ of standardized products. The price of customized products should not lower than that of standardized products in the retail channel.

**Assumption 3**. The demands of consumers in both channels are positive, *D*_*e*_ > 0 and *D*_*m*_ > 0.

**Assumption 4**. All input parameters are positive.

**Assumption 5**. θ (0 ≤ θ < 1) measures channel substitution. When θ approaches 1, the channel become perfect substitutes. The demand for each channel become independent when θ = 0.

**Assumption 6**. In the centralized customization and standardization channels, the following assumptions are established, $4k_{e}k_{m}\left(1-\theta^{2}\right)-2k_{e}{\lambda_{m}}^{2}-2k_{m}{\lambda_{e}}^{2}+{\lambda_{e}}^{2}{\lambda_{m}}^{2}>0$.

**Assumption 7**. In the decentralized customization and standardization channels, the following assumptions are established, $\text{2}\left(1-\theta^{2}\right)k_{e}-{\lambda_{e}}^{\text{2}}>0$ and $\frac{2Qk_{m}\left(\left(1-\theta^{2}\right)\left(2-M\lambda_{e}\right)\right)-{\left(Q\lambda_{m}\right)}^{2}}{{\left(1-\theta^{2}\right)}^{2}{\left(2-M\lambda_{e}\right)}^{2}}>0$.

**Assumption 8**. In the cost-sharing customization and standardization channels, the following assumptions are established, $\text{2}\left(1-\tau \right)\left(1-\theta^{2}\right)k_{e}-{\lambda_{e}}^{\text{2}}>0$.

### 3.1. The Centralized Dual Channel Supply Chain

We first consider the case of centralized decision. Both players in the supply chain act as a single system to maximize the whole supply chain profit. The manufacturer produces two products, a standardized product, sold through the retailer' channel and a customized product, sold through the customized channel. The decision variables are the price of customized products *p*_*m*_, the price of standardized products *p*_*e*_, the degree of customization *m* and the degree of standardization *e*. The total profit of the centralized dual channel supply chain is


(8) $$\begin{array}{l} \Pi_{T}\left(p_{e},p_{m},e,m\right)=\left(p_{e}-c_{e}\right)D_{e}+\left(p_{m}-c_{m}\right)D_{m}-\frac{K_{m}}{2}m^{2}-\frac{K_{e}}{2}e^{2} \end{array}$$

According to the above total profit function, we solve the first order partial derivatives with respect to *p*_*e*_, *p*_*m*_, *e* and *m*, respectively, and then we solve all of those equations simultaneously.


(9) $$\begin{array}{l} \{\begin{array}{c} \frac{\partial \Pi_{T}\left(p_{e},p_{m},e,m\right)}{\partial p_{e}}=0\\ \frac{\partial \Pi_{T}\left(p_{e},p_{m},e,m\right)}{\partial p_{m}}=0\\ \frac{\partial \Pi_{T}\left(p_{e},p_{m},e,m\right)}{\partial e}=0\\ \frac{\partial \Pi_{T}\left(p_{e},p_{m},e,m\right)}{\partial m}=0 \end{array} \end{array}$$

We can get the optimal degree of standardization is


(10) $$\begin{array}{l} e_{C}^{*}=\frac{-\theta \lambda_{e}\left(2k_{m}\right)\left(\alpha_{m}-c_{m}\right)+\lambda_{e}\left(2k_{m}-\lambda_{m}^{2}\right)\left(\alpha_{e}-c_{e}\right)}{4k_{m}k_{e}\left(1-\theta^{2}\right)-2k_{e}\lambda_{m}^{2}-2k_{m}\lambda_{e}^{2}+\lambda_{e}^{2}\lambda_{m}^{2}} \end{array}$$

The optimal degree of customization is


(11) $$\begin{array}{l} m_{C}^{*}=\frac{-\theta \lambda_{m}\left(2k_{e}\right)\left(\alpha_{e}-c_{e}\right)+\lambda_{m}\left(2k_{e}-\lambda_{e}^{2}\right)\left(\alpha_{m}-c_{m}\right)}{4k_{m}k_{e}\left(1-\theta^{2}\right)-2k_{e}\lambda_{m}^{2}-2k_{m}\lambda_{e}^{2}+\lambda_{e}^{2}\lambda_{m}^{2}} \end{array}$$

The optimal price of the standardized products is


(12) $$\begin{array}{l} p_{e-C}^{*}=\frac{-\theta \lambda_{e}^{2}\left(2k_{m}\right)\left(\alpha_{m}-c_{m}\right)+\left(4k_{m}k_{e}\left(1-\theta^{2}\right)-2k_{e}\lambda_{m}^{2}\right)\alpha_{e}}{2}\\ \;+\frac{\left(4k_{m}k_{e}\left(1-\theta^{2}\right)-2k_{e}\lambda_{m}^{2}-4k_{m}\lambda_{e}^{2}+2\lambda_{e}^{2}\lambda_{m}^{2}\right)c_{e}}{2} \end{array}$$

The optimal price of the customized products is


(13) $$\begin{array}{l} p_{m-C}^{*}=\frac{-\theta \lambda_{m}^{2}\left(2k_{e}\right)\left(\alpha_{e}-c_{e}\right)+\left(4k_{m}k_{e}\left(1-\theta^{2}\right)-2k_{m}\lambda_{e}^{2}\right)\alpha_{m}}{2}\\ \;+\frac{\left(4k_{m}k_{e}\left(1-\theta^{2}\right)-4k_{e}\lambda_{m}^{2}-2k_{m}\lambda_{e}^{2}+2\lambda_{e}^{2}\lambda_{m}^{2}\right)c_{m}}{2} \end{array}$$

We substitute (Equations 8, 10–13). We can get the Theorem 1.

**Theorem 1**. The optimal profit under the centralized case is


$$\begin{array}{l} \begin{array}{l} \pi_{T-C}^{*}=\left(p_{e-C}^{*}-c_{e}\right)\frac{\alpha_{e}-\theta \alpha_{m}-p_{e-C}^{*}+\theta p_{m-C}^{*}+\lambda_{e}e_{C}^{*}-\theta \lambda_{m}m_{C}^{*}}{1-\theta^{2}}\\ \;+\left(p_{m-C}^{*}-c_{m}\right)\frac{\alpha_{m}-\theta \alpha_{e}-p_{m-C}^{*}+\theta p_{e-C}^{*}+\lambda_{m}m_{C}^{*}-\theta \lambda_{e}e_{C}^{*}}{1-\theta^{2}}\\ \;-\frac{k_{m}{m_{C}^{*}}^{2}}{2}-\frac{k_{e}{e_{C}^{*}}^{2}}{2} \end{array} \end{array}$$

### 3.2. The Decentralized Dual Channel Supply Chain

In the decentralized case, the manufacturer and the retailer take into account their own profit maximization for the decision making. The retailer has to compete with the customized channel owned by the manufacturer.

The manufacturer's profit function is


(14) $$\begin{array}{l} \Pi_{M}\left(p_{m},m\right)=\left(w-c_{e}\right)D_{e}+\left(p_{m}-c_{m}\right)D_{m}-\frac{k_{m}}{2}m^{2} \end{array}$$

The retailer's profit function is


(15) $$\begin{array}{l} \Pi_{R}\left(p_{e},e\right)=\left(p_{e}-w\right)D_{e}-\frac{k_{e}}{2}e^{2} \end{array}$$

We adopt a two-stage Stackelberg game between the manufacturer and the retailer. The following sequence of events occurs in the game.

1. The manufacturer is the leader and the retailer is the follower.
2. The manufacturer chooses the degree of customization and decides the price of the customized products.
3. The retailer determines the retail price for standardized products and the degree of standardization.

$$\begin{array}{l} \{\begin{array}{l} \frac{\partial \Pi_{R}\left(p_{e},e\right)}{\partial p_{e}}=\frac{\alpha_{e}-\theta \alpha_{m}-p_{e}+\theta p_{m}+\lambda_{e}e-\theta \lambda_{m}m}{1-\theta^{2}}+\frac{-\left(p_{e}-\omega \right)}{1-\theta^{2}}=0\\ \frac{\partial \Pi_{R}\left(p_{e},e\right)}{\partial e}=\frac{\lambda_{e}\left(p_{e}-\omega \right)}{1-\theta^{2}}-k_{e}e=0 \end{array} \end{array}$$

Therefore,


(16) $$\begin{array}{l} \{\begin{array}{l} p_{e-E}=\frac{\alpha_{e}-\theta \alpha_{m}+\theta p_{m}-M\lambda_{e}\omega -\theta \lambda_{m}m+\omega }{2-M\lambda_{e}}\\ e_{E}=M\left(\frac{\alpha_{e}-\theta \alpha_{m}+\theta p_{m}-M\lambda_{e}\omega -\theta \lambda_{m}m+\omega }{2-M\lambda_{e}}-\omega \right) \end{array} \end{array}$$

We extend the functions of *D*_*e*_ and *D*_*m*_ as follows.


(17) $$\begin{array}{l} D_{e-E}=\frac{\left\{\begin{array}{c} \alpha_{e}-\theta \alpha_{m}-\frac{\alpha_{e}-\theta \alpha_{m}+\theta p_{m}-M\lambda_{e}\omega -\theta \lambda_{m}m+\omega }{2-M\lambda_{e}}+\theta p_{m}\\ +\lambda_{e}M\left(\frac{\alpha_{e}-\theta \alpha_{m}+\theta p_{m}-M\lambda_{e}\omega -\theta \lambda_{m}m+\omega }{2-M\lambda_{e}}-\omega \right)-\theta \lambda_{m}m \end{array}\right\}}{1-\theta^{2}} \end{array}$$

(18) $$\begin{array}{ll} D_{m-E}= & \frac{\left\{\begin{array}{c} \alpha_{m}-\theta \alpha_{e}-p_{m}+\theta \frac{\alpha_{e}-\theta \alpha_{m}+\theta_{p_{m}}-M\lambda_{e}\omega -\theta \lambda_{m}m+\omega }{2-M\lambda_{e}}+\lambda_{m}m\\ -\theta \lambda_{e}A\left(\frac{\alpha_{e}-\theta \alpha_{m}+\theta p_{m}-M\lambda_{e}\omega -\theta \lambda_{m}m+\omega }{2-M\lambda_{e}}-\omega \right) \end{array}\right\}}{1-\theta^{2}} \end{array}$$

Equations (16)–(18) are introduced to Equation (14), we get the maximum value


(19) $$\begin{array}{l} \{\begin{array}{l} p_{m-E}^{*}=\frac{\alpha_{m}}{2}+\frac{\lambda_{m}\left[\left(\omega -c_{e}\right)\frac{-2\theta \lambda_{m}}{2Nk_{m}-Q\lambda_{m}^{2}}+\left(\alpha_{m}+\frac{-\theta \alpha_{e}+\left(\theta +\theta M\lambda_{e}\right)\omega -\theta c_{e}}{Q}-c_{\text{m}}\right)\frac{Q\lambda_{m}}{2Nk_{m}-Q\lambda_{m}^{2}}\right]}{2}\\ \;+\frac{-\theta \alpha_{e}+\left(\theta +\theta M\lambda_{e}\right)\omega -\theta c_{e}}{2Q}+\frac{c_{\text{m}}}{2}\\ m_{E}^{*}\;=\left(\omega -c_{e}\right)\frac{-2\theta \lambda_{m}}{2Nk_{m}-Q\lambda_{m}^{2}}+\left(\alpha_{m}+\frac{-\theta \alpha_{e}+\left(\theta +\theta M\lambda_{e}\right)\omega -\theta c_{e}}{Q}-c_{\text{m}}\right)\frac{Q\lambda_{m}}{2Nk_{m}-Q\lambda_{m}^{2}} \end{array} \end{array}$$

It follows that


(20) $$\begin{array}{l} \{\begin{array}{ll} p_{e-E}^{*} & =\frac{\alpha_{e}-\theta \alpha_{m}+\theta p_{m-E}^{*}-M\lambda_{e}\omega -\theta \lambda_{m}m_{E}^{*}+\omega }{2-M\lambda_{e}}\\ e_{E}^{*} & =M\left(\frac{\alpha_{e}-\theta \alpha_{m}+\theta p_{m-E}^{*}-\theta \lambda_{m}m_{E}^{*}-\omega }{2-M\lambda_{e}}\right)\\ D_{e-E}^{*} & =\frac{\alpha_{e}-\theta \alpha_{m}-p_{e-E}^{*}+\theta p_{e-E}^{*}+\lambda_{e}e_{E}^{*}-\theta \lambda_{m}m_{E}^{*}}{1-\theta^{2}}\\ D_{m-E}^{*} & =\frac{\alpha_{m}-\theta \alpha_{e}-p_{m-E}^{*}+\theta p_{e-E}^{*}+\lambda_{m}m_{E}^{*}-\theta \lambda_{e}e_{E}^{*}}{1-\theta^{2}} \end{array} \end{array}$$

where $M=\frac{\lambda_{e}}{\left(1-\theta^{2}\right)k_{e}},N=\left(1-\theta^{2}\right)\left(2-M\lambda \right),Q=\left(2-M\lambda_{e}-\theta^{2}+\theta^{2}M\lambda_{e}\right)$.

We substitute (Equations 19 and 20) to the manufacturer's profit function (Equation 14) and the retailer's profit function (Equation 15). We can get the following theorem 2.

**Theorem 2**. The optimal profit of the manufacturer is $\Pi_{M-E}^{*}=\left(w-c_{e}\right)D_{e-E}^{*}+\left(p_{m-E}^{*}-c_{m}\right)D_{m-E}^{*}-\frac{k_{m}m_{E}^{*2}}{2}$ and the optimal profit of the retailer is $\Pi_{R-E}^{*}=\left(p_{e-E}^{*}-w\right)D_{e-E}^{*}-\frac{k_{e}e_{E}^{*2}}{2}$.

We analyze the supply chain decisions under the centralized channel and decentralized channel scenarios. To draw our results, we compare the price, the degree of standardization and the degree of customization of the supply chain under different channel structures. The default values of our input parameters are *w* = 2.5, α_*e*_ = 10, θ = 0.5, *c*_*m*_ = 1.5, *c*_*e*_ = 1.5, *K*_*m*_ = 1, *K*_*e*_ = 1, λ_*e*_ = 0.25. We set α_*m*_ as the independent variable while xing the values of other parameters. λ_*m*_ is associated to λ_*e*_. The following propositions summarize the comparison results.

**Proposition 1**. In both scenarios, the base demand of dual channel supply chain will affect the degree of customization, the degree of standardization and the overall supply chain performance


$$\frac{\partial {p^{\text{*}}}_{e-C}}{\partial \alpha_{e}}>0,\frac{\partial {p^{\text{*}}}_{e-E}}{\partial \alpha_{e}}>0,\frac{\partial {p^{\text{*}}}_{m\text{-}C}}{\partial \alpha_{m}}>0,\frac{\partial {p^{\text{*}}}_{m\text{-}E}}{\partial \alpha_{m}}>0,$$

$$\frac{\partial e_{C}^{*}}{\partial \alpha_{e}}>0,\frac{\partial e_{E}^{*}}{\partial \alpha_{e}}>0,\frac{\partial m_{C}^{*}}{\partial \alpha_{m}}>0,\frac{\partial m_{E}^{*}}{\partial \alpha_{m}}>0$$

$$\frac{\partial e_{C}^{*}}{\partial \alpha_{m}}<0,\frac{\partial e_{E}^{*}}{\partial \alpha_{m}}<0,\frac{\partial m_{C}^{*}}{\partial \alpha_{e}}<0,\frac{\partial m_{E}^{*}}{\partial \alpha_{e}}<0$$

According to Equations (10)–(13), (19), and (20), we conclude that the optimal product pricing is related to the channel's potential market demand, whether we analyze the decentralized or the centralized case. The price of customized products will increase as the demand for customization increases, and the price of standardized products will increase as the demand for standardized products increases. The degree of customization and standardization between channels also changes according to the basic needs of the channel, for example, the degree of customization increases according to the basic needs of the customization channel. Channel managers need to work hard to explore the market potential customization needs and expand the customization market. In the field of security video surveillance, there are always some manufacturers will ignore the customer needs. In the era of network monitoring, it is essential for security companies to grasp the actual needs of customers and design appropriate customized products.

**Proposition 2**. The price of standardized products rises as the degree of standardization increases. With the increased degree of standardization, the price of standardized products under the centralized scenario is higher than that of standardized products under the decentralized one. In addition, the sales price of standardized products is more affected by the degree of standardization in the decentralized case.

**Figure 1:**
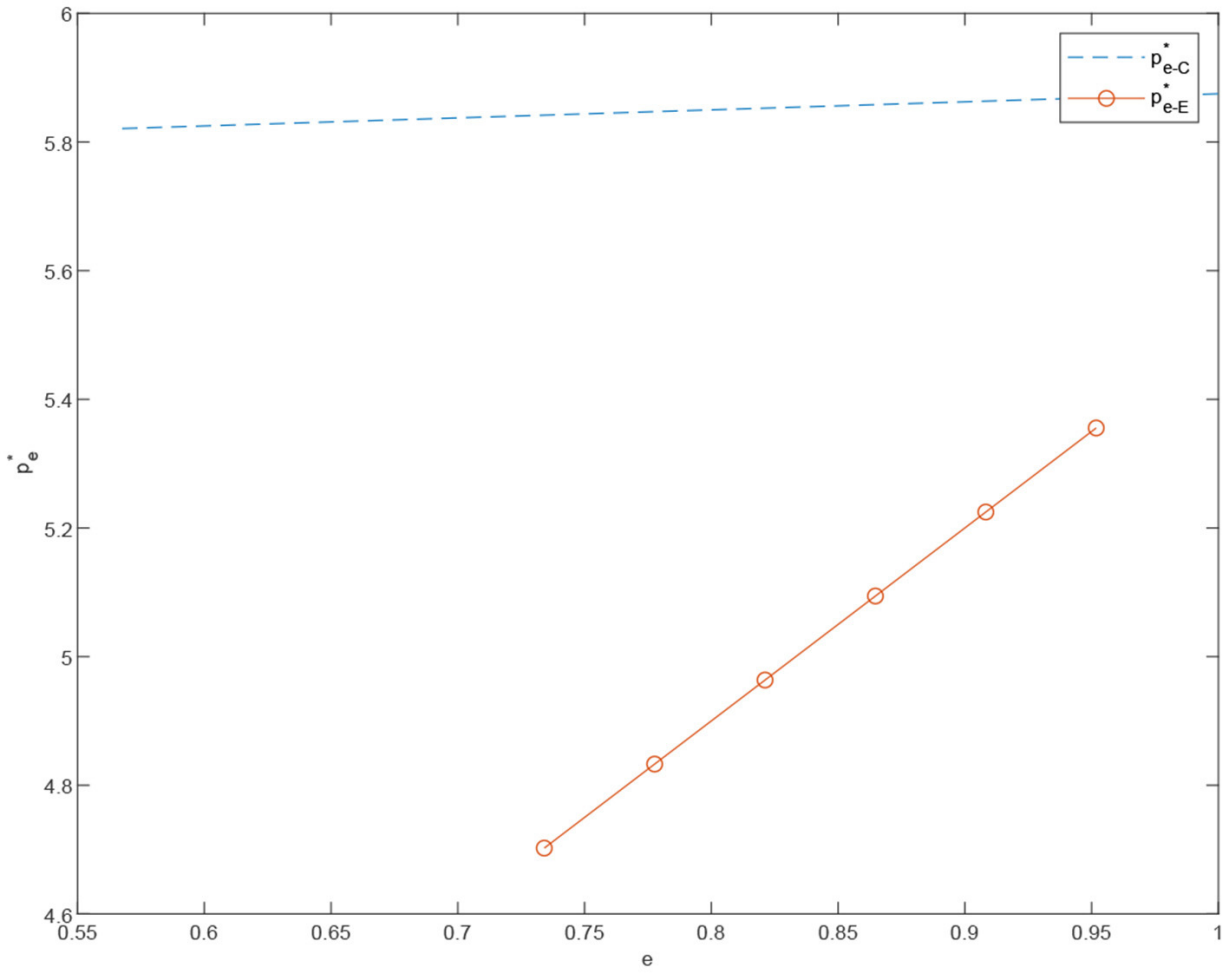
The price of the standardized product under the centralized scenario $p_{e-C}^{*}$ vs. the price of the standardized product under the decentralized scenario $p_{e-E}^{*}$.

Figure 1 indicates that in order to better take advantage of the standardized product channel, the retailer increase the investment in the construction of the standardized channel, which leads to an increase of the standardization price. Moreover, we find that under the decentralized case, the price of standardized products is more affected by the degree of standardization compared with the price of standardized products under the centralized case.

**Proposition 3**. In a dual channel supply chain, the price of the customized products rises as the degree of customization increases. More specifically, when the degree of customization *m* increases, the price of customized products in the centralized scenario is higher than that of customized products in the decentralized case, $p_{m-C}^{*}>p_{m-E}^{*}$.

**Figure 2:**
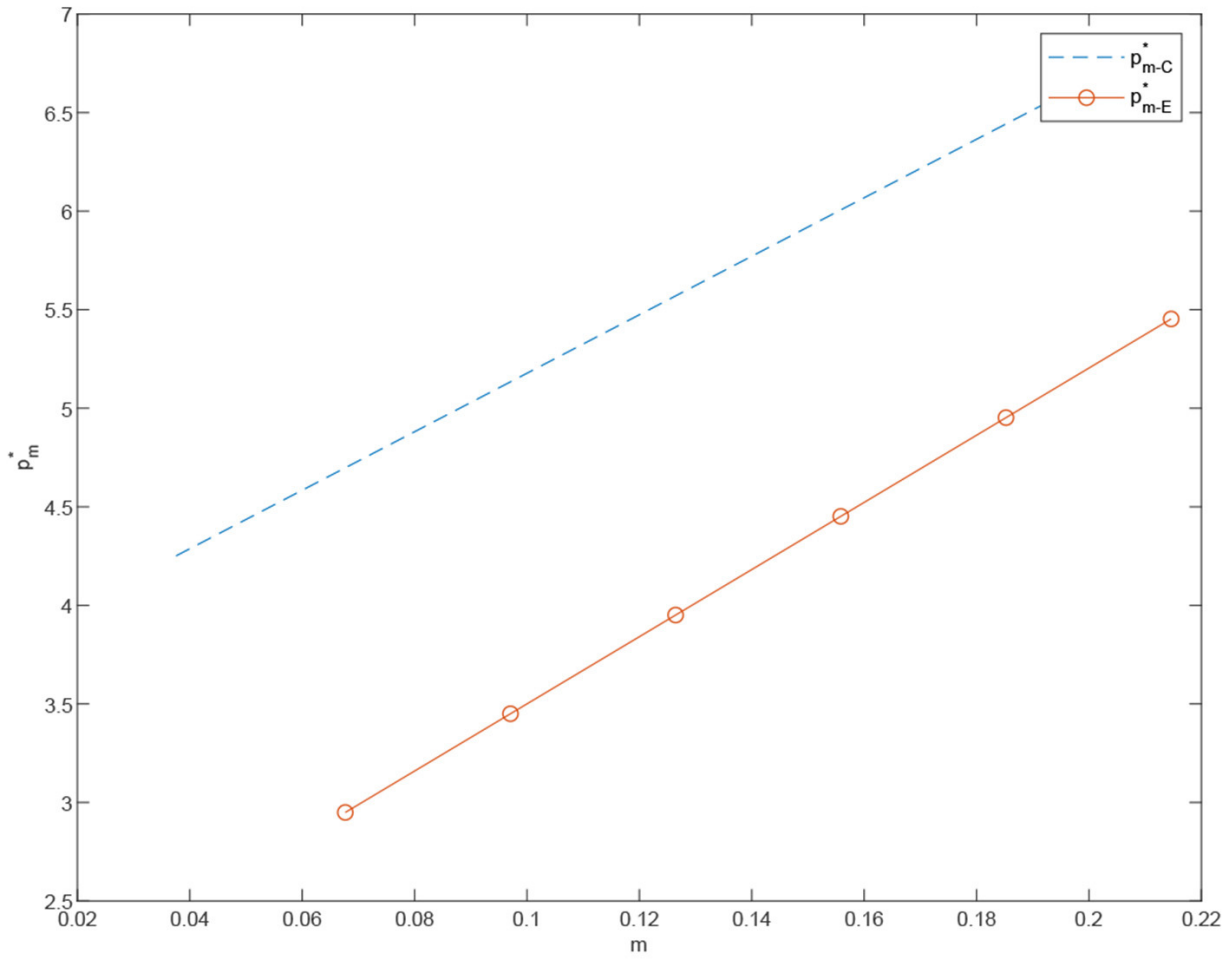
The price of the customized product under the centralized scenario $p_{m-C}^{*}$ vs. the price of the customized product under the decentralized scenario $p_{m-E}^{*}$.

Figure 2 indicates that to provide better customized products, the manufacturer increases the investment in both technical innovation and channel construction of customized products which includes the cost of building a customized platform, the cost of intelligent information production, and the cost of purchasing new equipment. As a result, the manufacturer increases the price of the customized products due to the increasing cost of customization.

**Figure 3:**
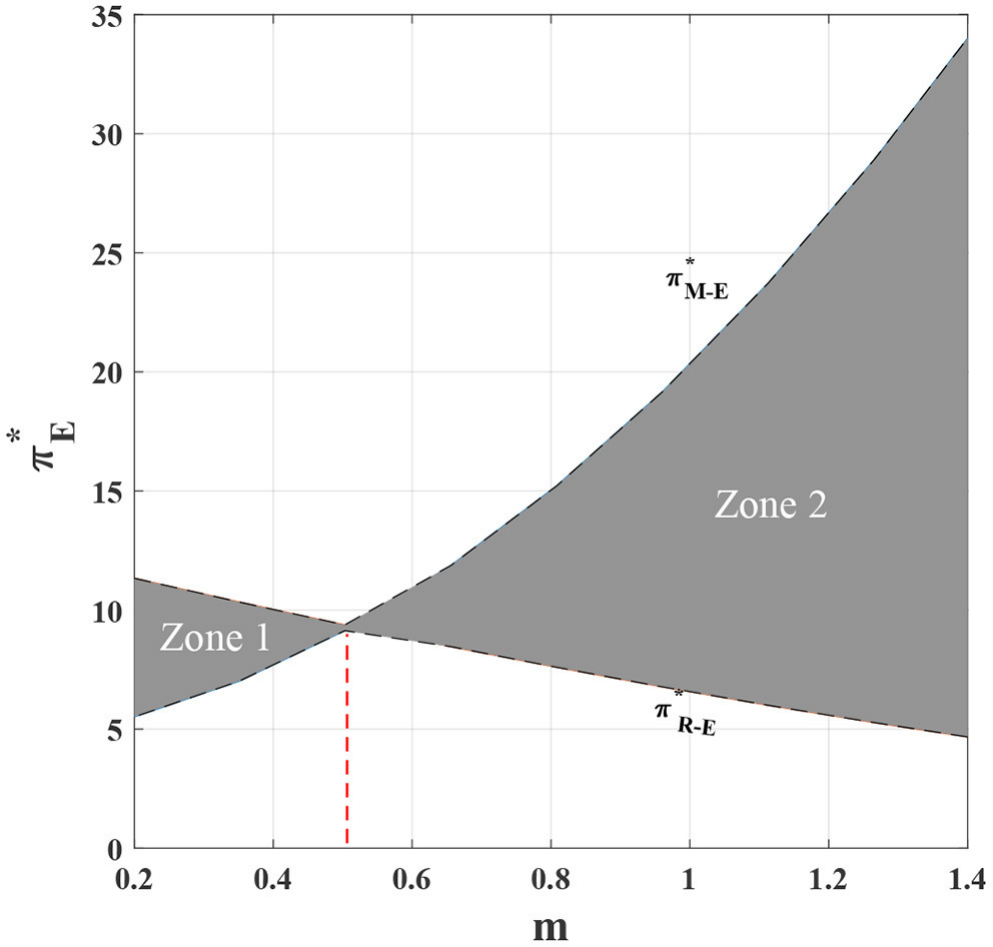
The impact of *m* on the channel profits under the decentralized cases.

Combining with Figure 3, it can be observed that under the decentralized scenario, the degree of customization has a significant impact on the channel profit. The profit of the manufacturer increases with the increase of the degree of customization, the profit of the retailer decreases with the increase of the degree of customization, and the manufacturer is more obviously affected by the degree of customization. In the area 1 of the figure, the manufacturer's profit is lower than the retailer's profit, and in the area 2, as the degree of customization exceeds a certain threshold, the manufacturer gradually reaps a higher level of profit than the retailer. Then we can see that, the manufacturer's profit from both the customized and standardized channels is greater than the retailer's profit from only the standardized channel. That is, $\Pi_{M-E}^{*}>\Pi_{R-E}^{*}$. According to the above analysis, it can be found that the introduction of customized channel is a good advantage for the manufacturer. By increasing the investment in customized channels, the level of channel customization can be continuously improved and a higher level of profit can be obtained. However, for the retailer, the customized channel compresses their original profit margins, which may lead to the retailer's dissatisfaction with the introduction of customized channel, which in turn leads to channel conflicts and imbalances in the system.

**Proposition 4**. In the decentralized dual channel supply chain, on the one hand, manufacturers' profits rise significantly with increasing channel customization, while on the other hand, retailers' profits fall steadily. More specifically, the manufacturer's profit increase is economically greater than the decline in the retailer's profit, therefore the overall supply chain profit is still increasing.

**Figure 4:**
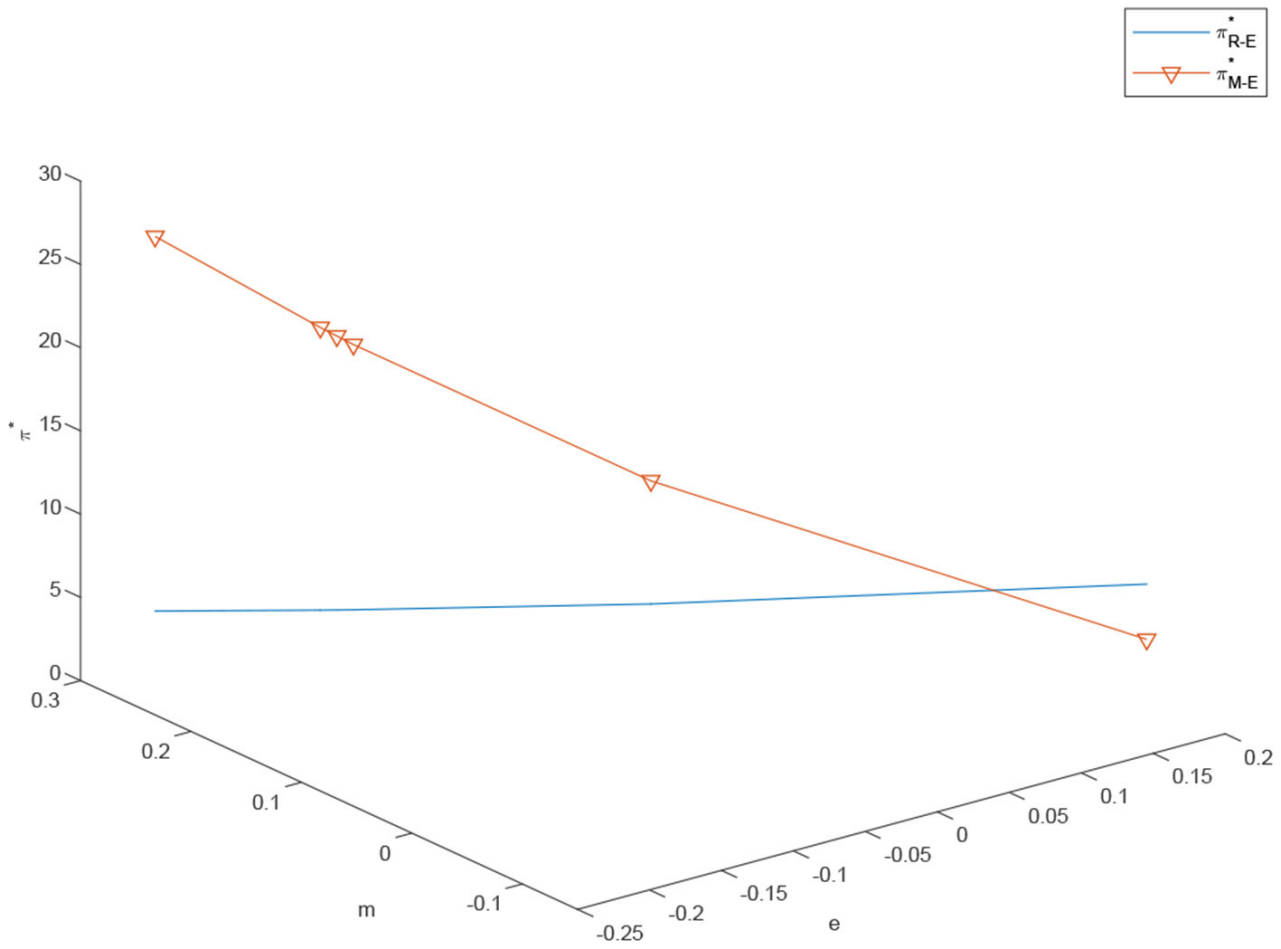
The effects of *e* and *m* on the retailer's and manufacturer's and profits under centralized scenario, $\pi_{R-E}^{*}$ and $\pi_{M-E}^{*}$.

In Figure 4, we observe that the increase in customization significantly increases the manufacturer's profitability. Conversely, the existence of channel conflicts leads to a considerable impact on the retailer's profitability. In the home appliance industry, the customization has become an important direction of transformation and upgrading.With young users gradually becoming the main body of household appliances end-consumers, more and more consumers want to be able to highlight their distinctive personality, the pursuit of technology, fashion, comfort and personalized life. As a consequence, companies such as Haier, Midea, LG, Samsung, Bosch, Siemens and many others have launched customized services for home appliance products to meet the customized needs of their consumers. With the gradual development of the customization market, the original standardized home appliance market is bound to shrink resulting in the overall decline of the standardized market profit. 

**Proposition 5**. We find that the introduction of a customized channel increases the total profit of the system, especially as the market becomes more customizable.

**Figure 5:**
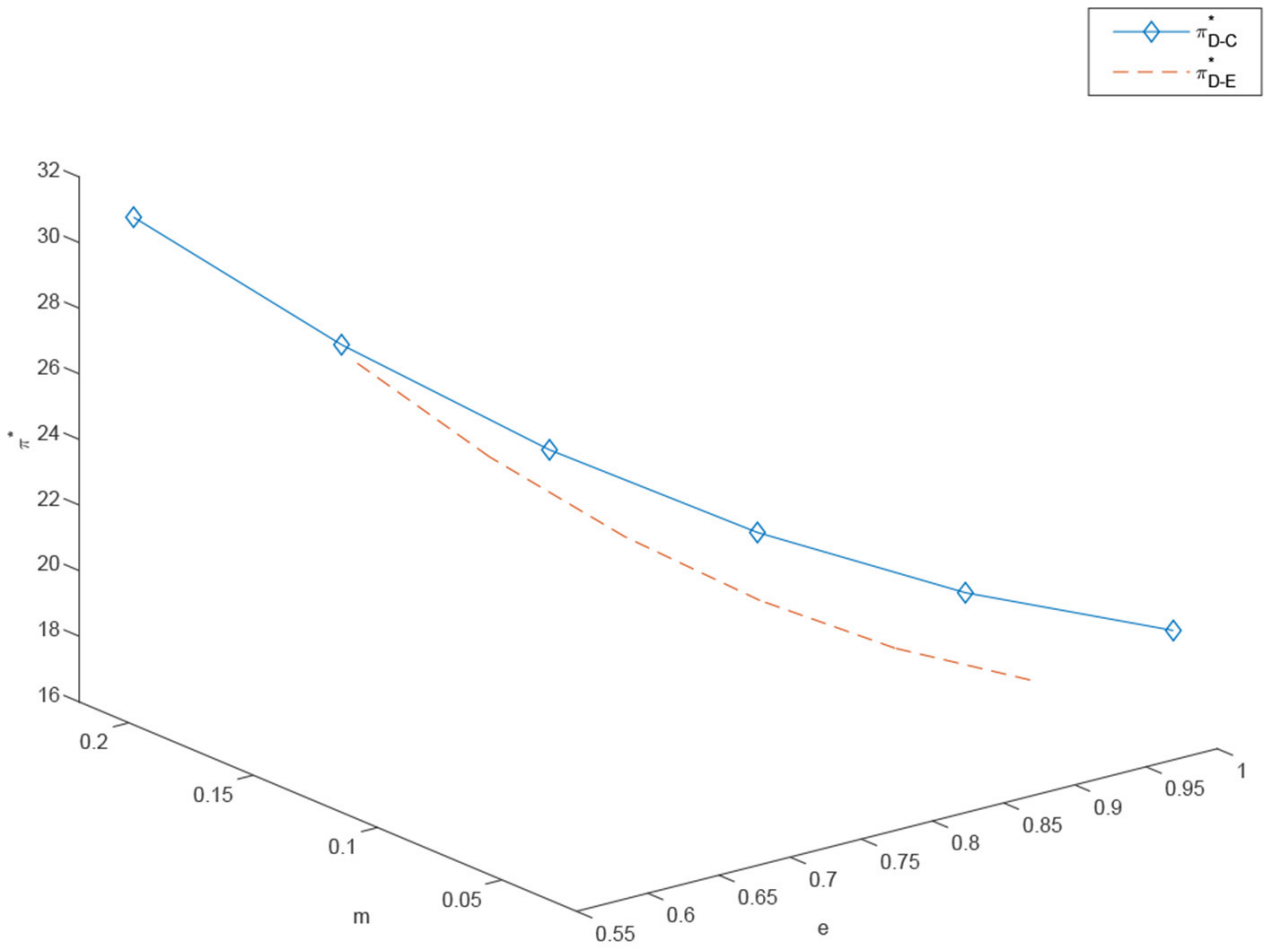
The effects of *e* and *m* on the retailer's and manufacturer's and profits under decentralized scenario, $\pi_{D-C}^{*}$ and $\pi_{D-E}^{*}$.

Figure 5 indicates that as the degree of customization *m* increases, the degree of standardization *e* decreases somewhat, but the total profit of the system increases significantly. To conclude, the channel manager should strive to introduce a customized channel or, if already existing, increase the research and development capital investments in it. Furthermore, the channel manager should do everything in the power to coordinate the supply chain and centralize the supply chain system to maximize the company's total profit. Channel managers should establish the optimal balance between the benefits of investing and increasing the customized channel, and the price increase derived by such investment. More precisely, adding a customized channel is in the manufacturer's best interest only when the customized market demand is large enough to economically justify the capital investment. For this reason, in recent years, more and more industries have chosen to add a customized channel. However, when the market demand for customization is sluggish, choosing to add a customized channel does not only directly decrease the retailer's profit and cause a channel conflict, but also fails to increase the profit of the manufacturer. This also corresponds to practice explaining why many industries do not have the option of customization, such as electric cars and hair dryers.

Because of the strong dependence of the manufacturer's choice to invest in new customized channels on the level of customized products demand, we consider the influence of the standardized elasticity of demand and the customized elasticity of demand on the supply chain decision making. Due to the incomplete substitutability between customized and standardized products, customers are heterogeneous in their preference between them. As a result, the consumers' purchase behaviors for both products lead to different channel influence coefficients between the two channels.

**Figure 6:**
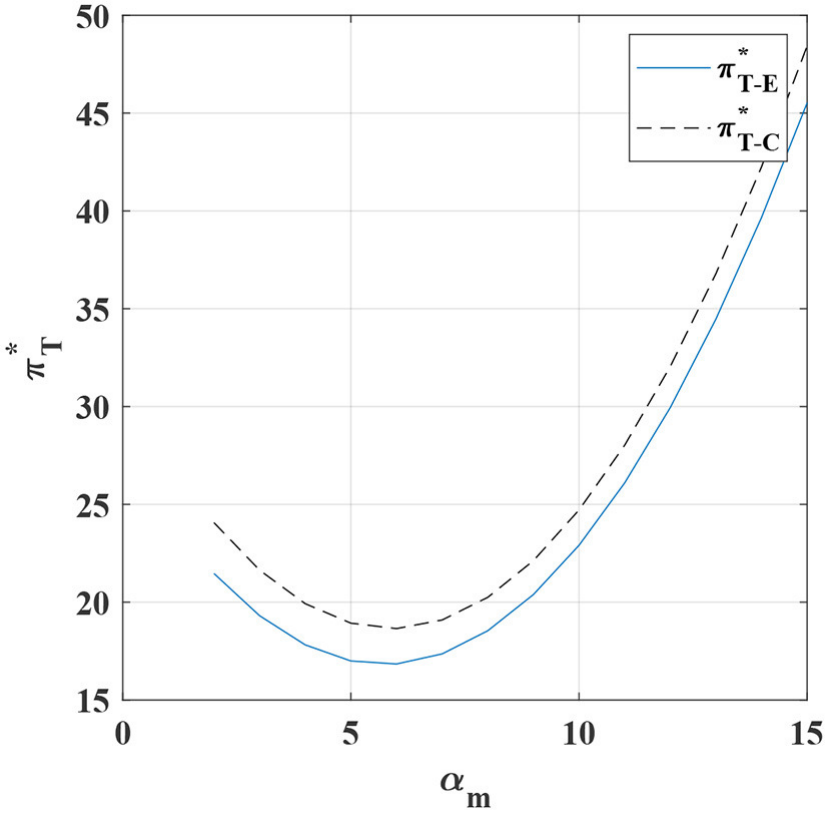
The impact of α_*m*_ on the overall profit of the system under the different cases.

Equations (19) and (20) show that the degree of customization between those channels and the customized market demand, as well as the degree of standardization and the standardized market demand, also show a positive correlation. For instance, the degree of customization will increase according to the increase in the customized market demand. The same trend also applies to the standardized market. However, as shown in Figure 6, there is no linear growth relationship between the customized market demand and the overall profit of the system. Specifically, when the market demand for customization is large, adding a customized channel is a good choice for the system. Because even if there are higher fixed costs of production technology and higher marginal production costs, manufacturers can respond faster to customer needs by increasing customized channels, enhance channel competitiveness, and gain more profits. This also explains why in recent years, more and more industries have begun to choose to add a customized channel. However, when the market demand for customization is sluggish, choosing to add a customized channel is not only not cost-effective or flexible, but also affects the profits of the manufacturer and the retailer, and is not conducive to the long-term stability of the system.

To quantify the differences, we use the parameters λ_*m*_ to reflect the effect of an increase in the degree of customization and standardization on dual channel's demand, respectively. We use λ_*r*_ to reflect the effect of an increase in the degree of standardization on dual channel's demand. Let λ_*m*_ = φλ_*r*_(*A* ≥ 0), then φ measure the difference of coefficient between customization and standardization channel. When 0 < φ < 1, the customization channel has a weaker channel advantage. When 1 < φ, the customization channel has a stronger channel advantage. We use a numerical approach to study the effects of these parameters.

**Proposition 6**. In the centralized case, as φ increases, the customized elasticity of demand λ_*m*_ increases. The sales price of customized products presents an upward trend, and the sales price of standardized products remains relatively stable. Additionally, the growth rate of customization demand is growing at a greater rate than that of standardization demand. As a result, the supply chain systems achieve higher profits. In the decentralized case, the system profit increases in λ_*m*_, but the growth trend is not obvious.


$$\frac{\partial {D^{*}}_{m\text{-}E}}{\partial m}>\frac{\partial {D^{*}}_{\text{e -}E}}{\partial e},\frac{\partial {D^{*}}_{m\text{- C}}}{\partial m}>\frac{\partial {D^{*}}_{\text{e - C}}}{\partial e}$$

**Figure 7:**
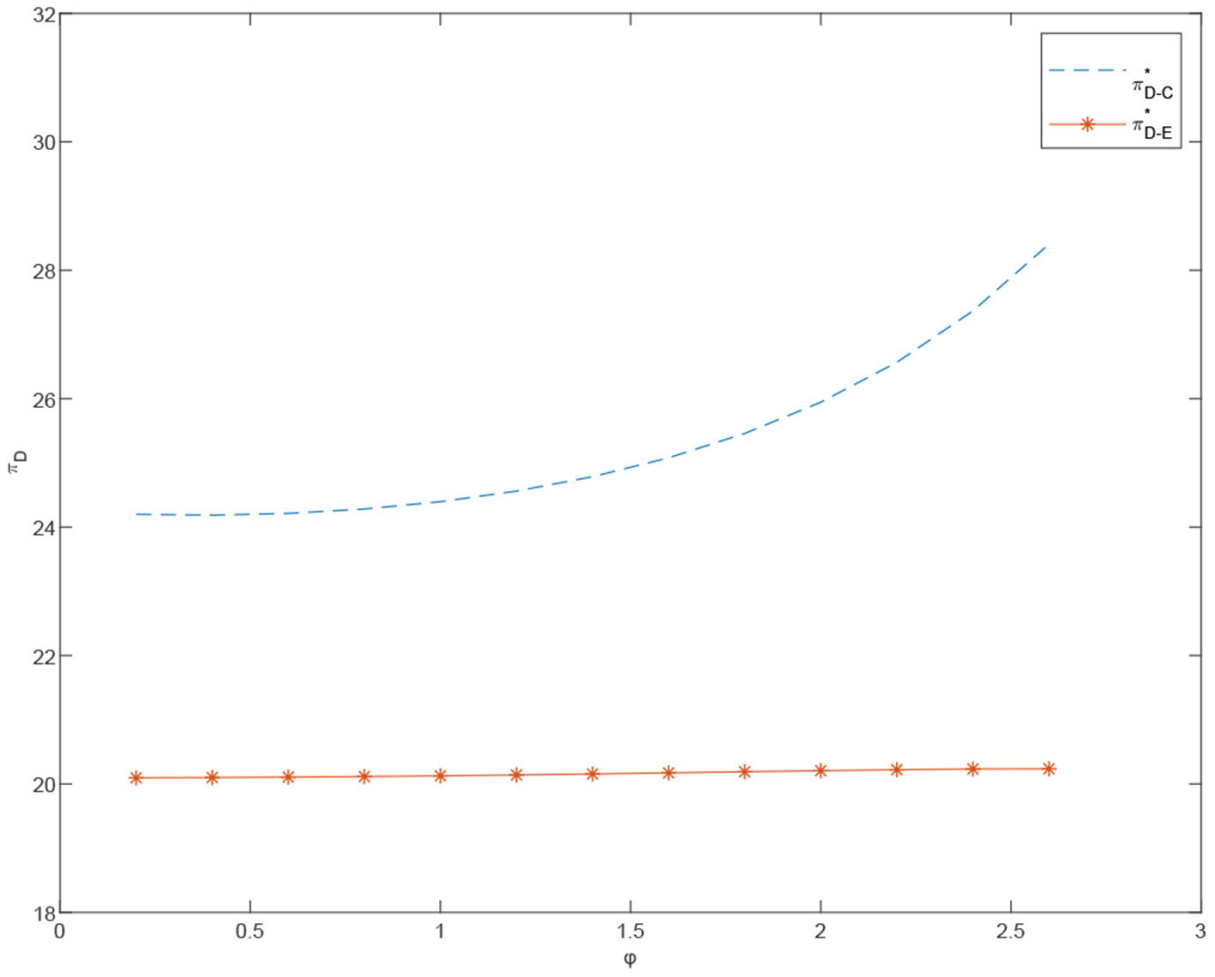
The effects of φ on the total profits under centralized and decentralized scenarios, $\pi_{T-C}^{*}$ and $\pi_{T-E}^{*}$.

**Figure 8:**
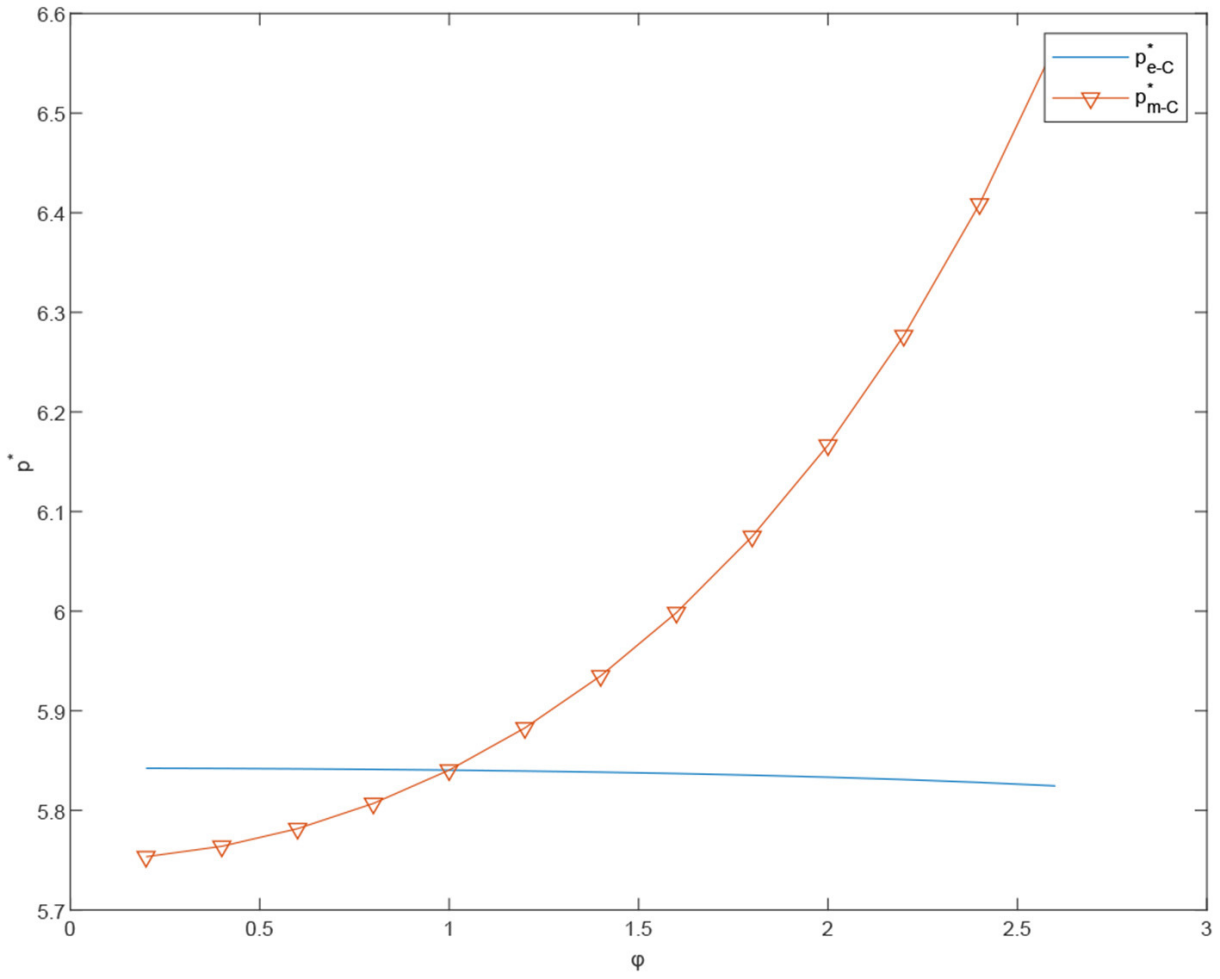
The effects of φ on the prices under the centralized scenario, $p_{e-C}^{*}$ and $p_{m-C}^{*}$.

Figures 7, 8 show that, in the centralized case, the customized channel profit increases with λ_*m*_. More specifically, the price of customized products continues to rise, while the price of standardized products remains relatively stable. The demand growth rate of customized products is steeper than that of standardized products and the total system profit keeps growing. On the other hand, in the decentralized case, overall channel profit does not increase significantly in λ_*m*_. This may be due to the channel conflict between the customized and the standardized channels.

## 4. Coordination Mechanism

Base on the above analysis, we conclude the total profit of the centralized supply chain is greater than that of the decentralized one. To improve the overall efficiency of the supply chain and eliminate the double marginal effect, we introduce a cost-sharing contract, in which the manufacturer and the retailer share the cost of standardized production.

Our aim is to reduce the conflict between the two channels. The manufacturer absorbs the standardized cost of $\tau \frac{k_{e}e^{2}}{2}$, and the retailer is responsible for the standardized cost of $\left(1-\tau \right)\frac{k_{e}e^{2}}{2}$.

The manufacturer's profit function under the cost-sharing contract is


(21) $$\begin{array}{l} \pi_{M}=\left(\omega -c_{\text{e}}\right)D_{\text{e}}+\left(p_{m}-C_{m}\right)D_{m}-\frac{k_{m}m^{2}}{2}-\tau \frac{k_{\text{e}}e^{2}}{2} \end{array}$$

The retailer's profit function under the cost-sharing contract is


(22) $$\begin{array}{l} \pi_{R}=\left(p_{\text{e}}-\omega \right)D_{\text{e}}-\left(1-\tau \right)\frac{k_{\text{e}}e^{2}}{2} \end{array}$$

In order to ensure the supply chain achieves an optimal coordination, the fllowing condition has to hold, $\pi_{D-C}^{*}>\pi_{D-O}^{*}$,$\pi_{D-C}^{*}>\pi_{D-E}^{*}$,$\pi_{D-O}^{*}>\pi_{D-E}^{*}$.

Within the coordination case of cost-sharing, we adopt a two-stage Stackelberg game in which the following sequence of events occurs.

At the first stage, the manufacturer decides the degree of customization and the price of customized products. In the second stage, the retailer decides the retail price of standardized products and the degree of standardization of the standardized channel. The reverse solution method can be used to obtain the price of the customized products, the price of the standardized products, the degree of customization and the degree of standardization. Under the cost-sharing contract, the optimal results are as follows.

The optimal degree of customization is


(23) $$\begin{array}{l} m_{O}^{*}=\left(\omega -c_{e}\right)\frac{-2\theta \lambda_{m}}{2Ek_{m}-F\lambda_{m}^{2}}\\ \;+\left(\alpha_{m}+\frac{-\theta \alpha_{e}+\left(\theta +\theta A\lambda_{e}\right)\omega -\theta c_{e}}{F}-c_{\text{m}}\right)\frac{F\lambda_{m}}{2Ek_{m}-F\lambda_{m}^{2}} \end{array}$$

The optimal price of the customized products is


(24) $$\begin{array}{l} \begin{array}{l} p_{m-O}^{*}=\frac{\alpha_{m}}{2}\\ +\frac{\lambda_{m}\left[\left(\omega -c_{e}\right)\frac{-2\theta \lambda_{m}}{2Ek_{m}-F\lambda_{m}^{2}}+\left(\alpha_{m}+\frac{-\theta \alpha_{e}+\left(\theta +\theta A\lambda_{e}\right)\omega -\theta c_{e}}{F}-c_{\text{m}}\right)\frac{F\lambda_{m}}{2Ek_{m}-F\lambda_{m}^{2}}\right]}{2}\\ +\frac{-\theta \alpha_{e}+\left(\theta +\theta A\lambda_{e}\right)\omega -\theta c_{e}}{2F}+\frac{c_{\text{m}}}{2} \end{array} \end{array}$$

The optimal degree of standardization is


(25) $$\begin{array}{l} e_{O}^{*}=\frac{\alpha_{e}-\theta \alpha_{m}+\theta p_{m-O}^{*}-\theta \lambda_{m}m_{O}^{*}-\omega }{2-A\lambda_{e}} \end{array}$$

The optimal price of the standardized products is


(26) $$\begin{array}{l} p_{e-O}^{*}=\frac{\alpha_{e}-\theta \alpha_{m}+\theta p_{m-O}^{*}-A\lambda_{e}\omega -\theta \lambda_{m}m_{O}^{*}+\omega }{2-A\lambda_{e}} \end{array}$$

where $A=\frac{\lambda_{e}}{\left(1-\theta^{2}\right)\left(1-\tau \right)k_{e}},E=\left(1-\theta^{2}\right)\left(2-A\lambda \right),F=\left(2-A\lambda_{e}-\theta^{2}+\theta^{2}A\lambda_{e}\right)$. To verify the validity of the mode, according to the default parameter settings, we imposed, 0 ≤ τ < 1. We substitute (Equations 23–26) to the manufacturer's profit function (Equation 21) and the retailer's profit function (Equation 22), to obtain the maximum value.

**Theorem 3**. The manufacturer's optimal profit is, $\pi_{M-O}^{*}=\left(\omega -c_{e}\right)D_{e-O}^{*}+\left(p_{m-O}^{*}-c_{m}\right)D_{m-O}^{*}-\frac{k_{m}m_{O}^{*2}}{2}-\frac{\tau k_{e}e_{O}^{*2}}{2}.$ The retailer's optimal profit is, $\pi_{R-O}^{*}=\left(p_{e-O}^{*}-\omega \right)D_{e-O}^{*}-\frac{\left(1-\tau \right)k_{e}e_{O}^{*2}}{2}.$

**Proposition 7**. The total profit of the coordinated dual channels is greater than that of the dual channels under the decentralized case when τ = 0.4 and α_*m*_ = 10. Moreover, as shown in Figure 8, with φ and λ_*m*_ constantly increasing, the total profit of the dual channel after coordination shows steadier growth, compared with the systematic profit under the decentralized case. This indicates that, after the coordination of cost-sharing contract, the supply chain decision making is less affected by the difference between λ_*m*_ and λ_*e*_.

**Proposition 8**. Comparing the customized product prices of centralized channels and decentralized channels, standardized product prices and customized product prices of cost-sharing channels, we can get the sales price under coordination mode when α_*m*_ = 7 and φ = 2.6 lower than the selling price under centralized decision-making. The sales price under coordinated mode is close to that under decentralized decision-making.

**Proposition 9**. In coordinated case, the price of standardized products *p*_*e*_ decreases in α_*m*_ and increases in τ. On the contrary, the price of customized products *p*_*m*_ will increase significantly in α_*m*_, but decrease slightly by τ.

**Figure 9:**
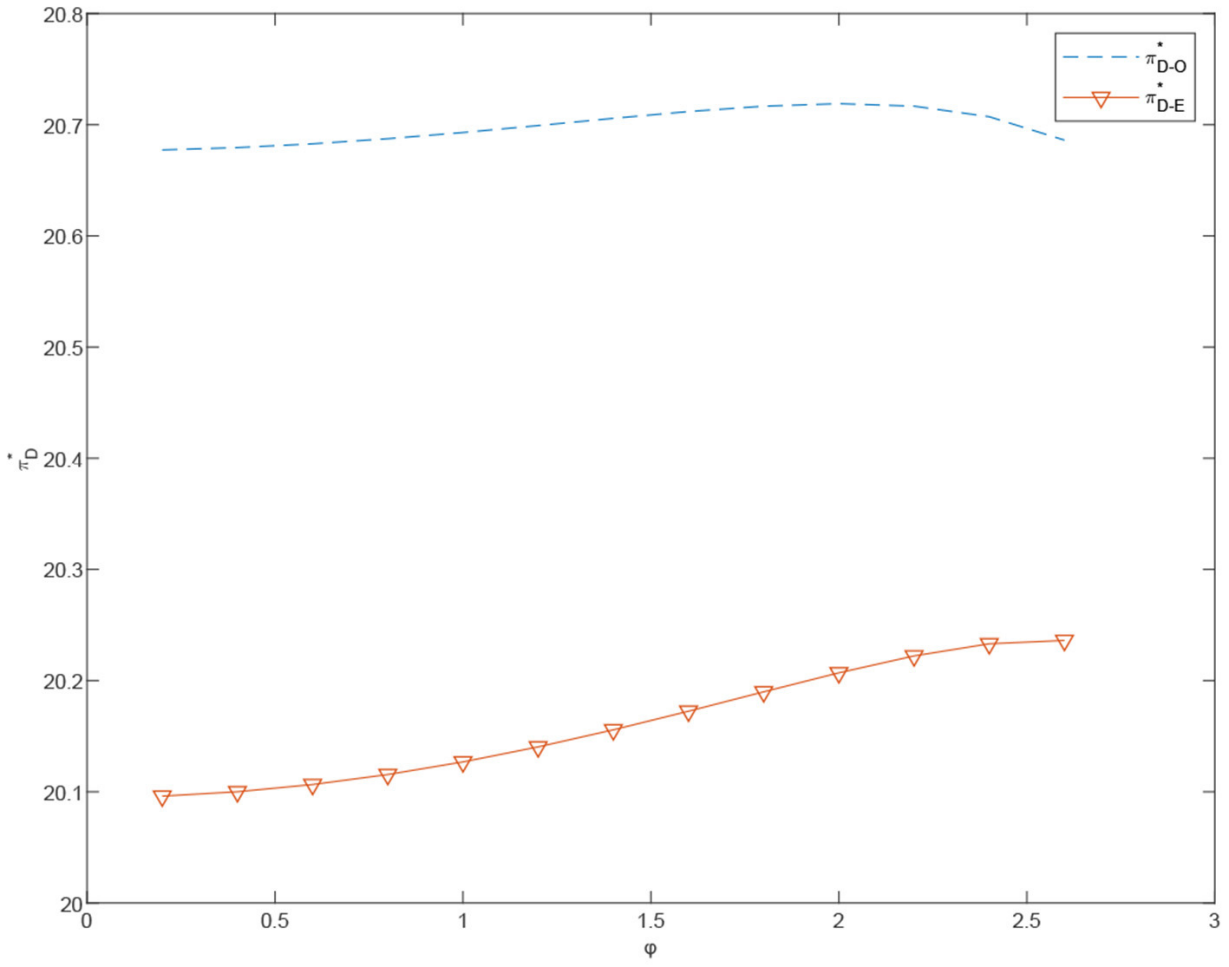
The effects of φ on the prices under the decentralized scenario, $\pi_{D-E}^{*}$ and $\pi_{D-O}^{*}$.

**Figure 10:**
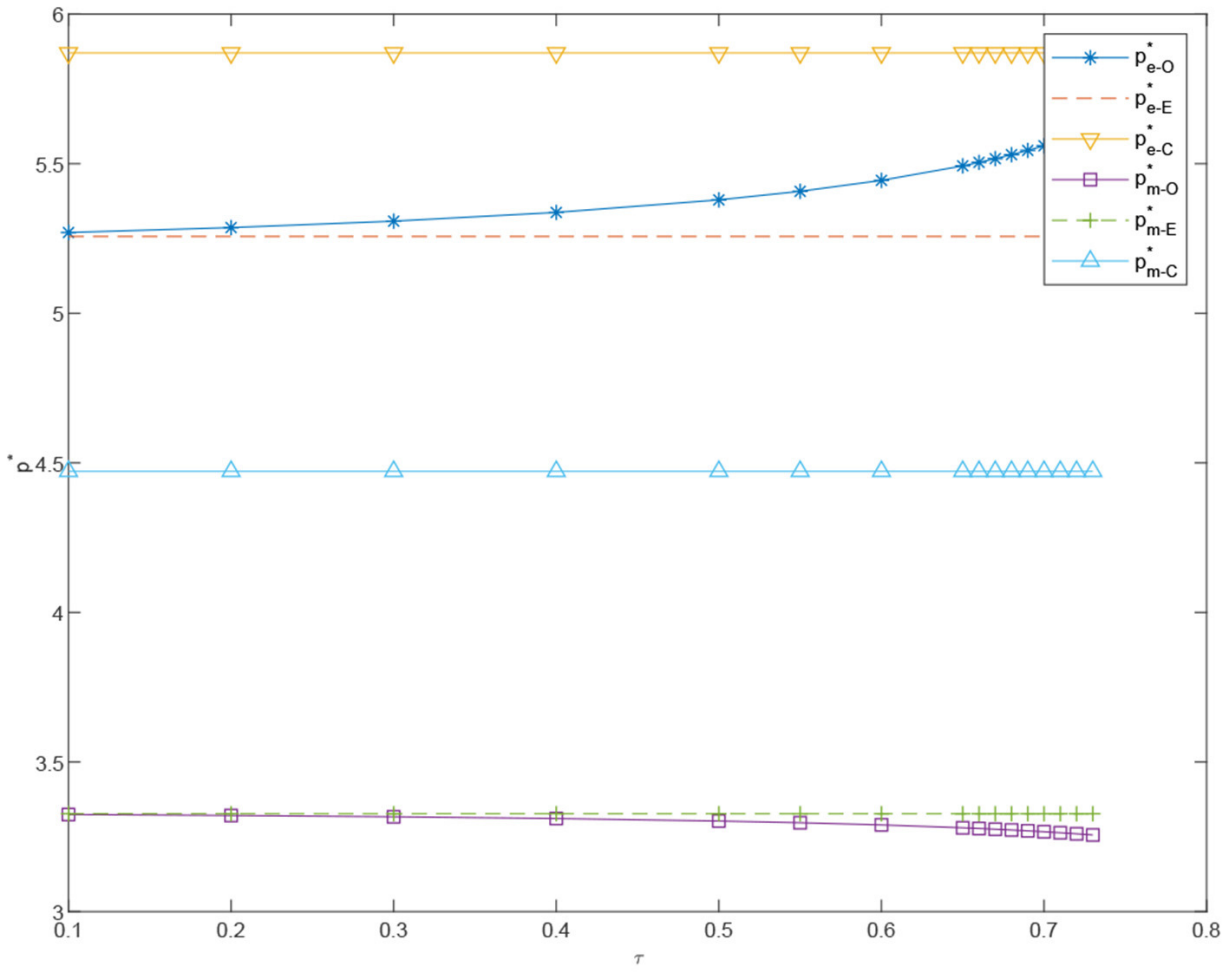
The effect of τ on the prices, $p_{e-O}^{*}$, $p_{e-E}^{*}$, $p_{e-C}^{*}$, $p_{m-O}^{*}$, $p_{m-E}^{*}$,$p_{m-C}^{*}$.

**Figure 11:**
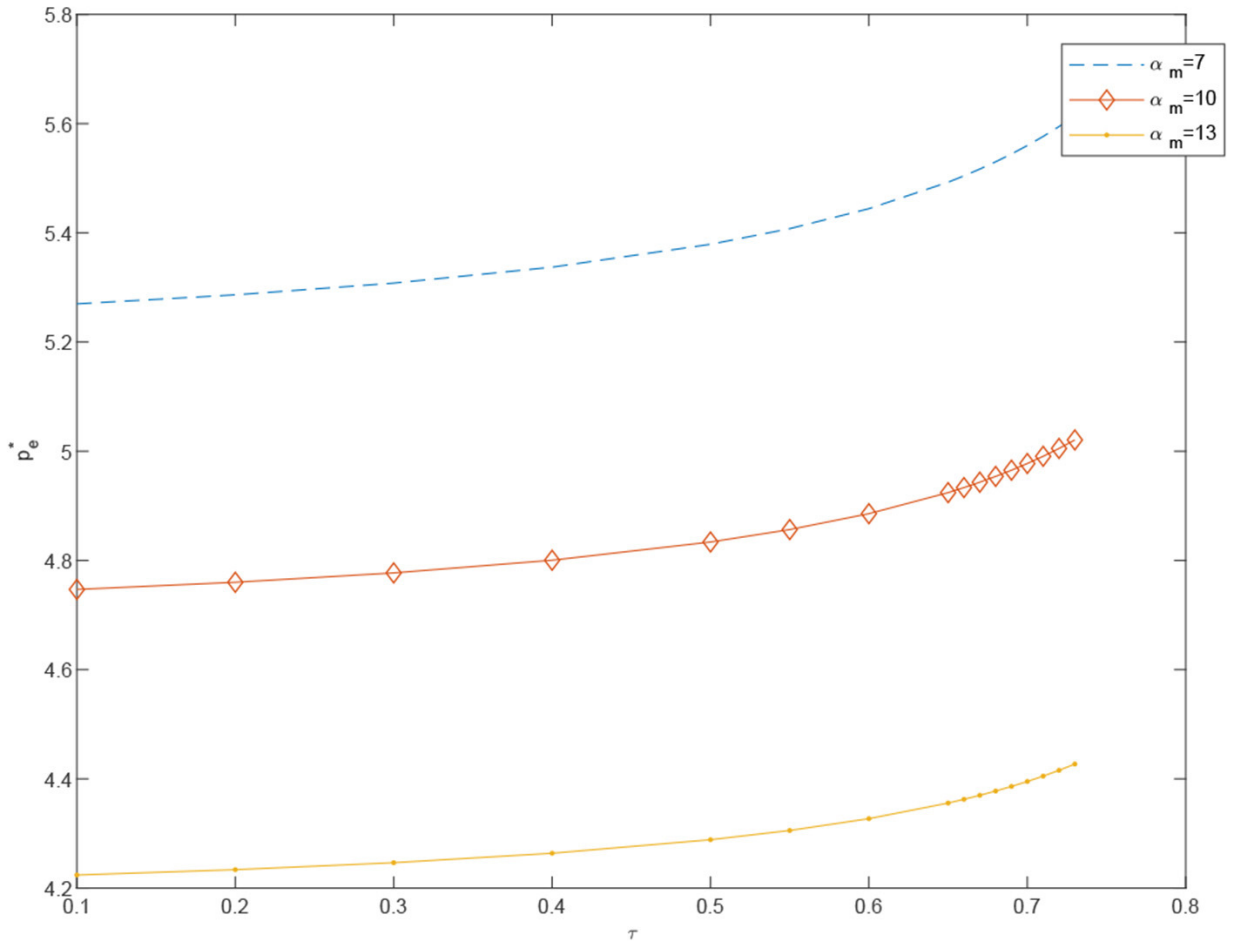
The effect of τ on $p_{e-O}^{*}$ under different α_*m*_.

**Figure 12:**
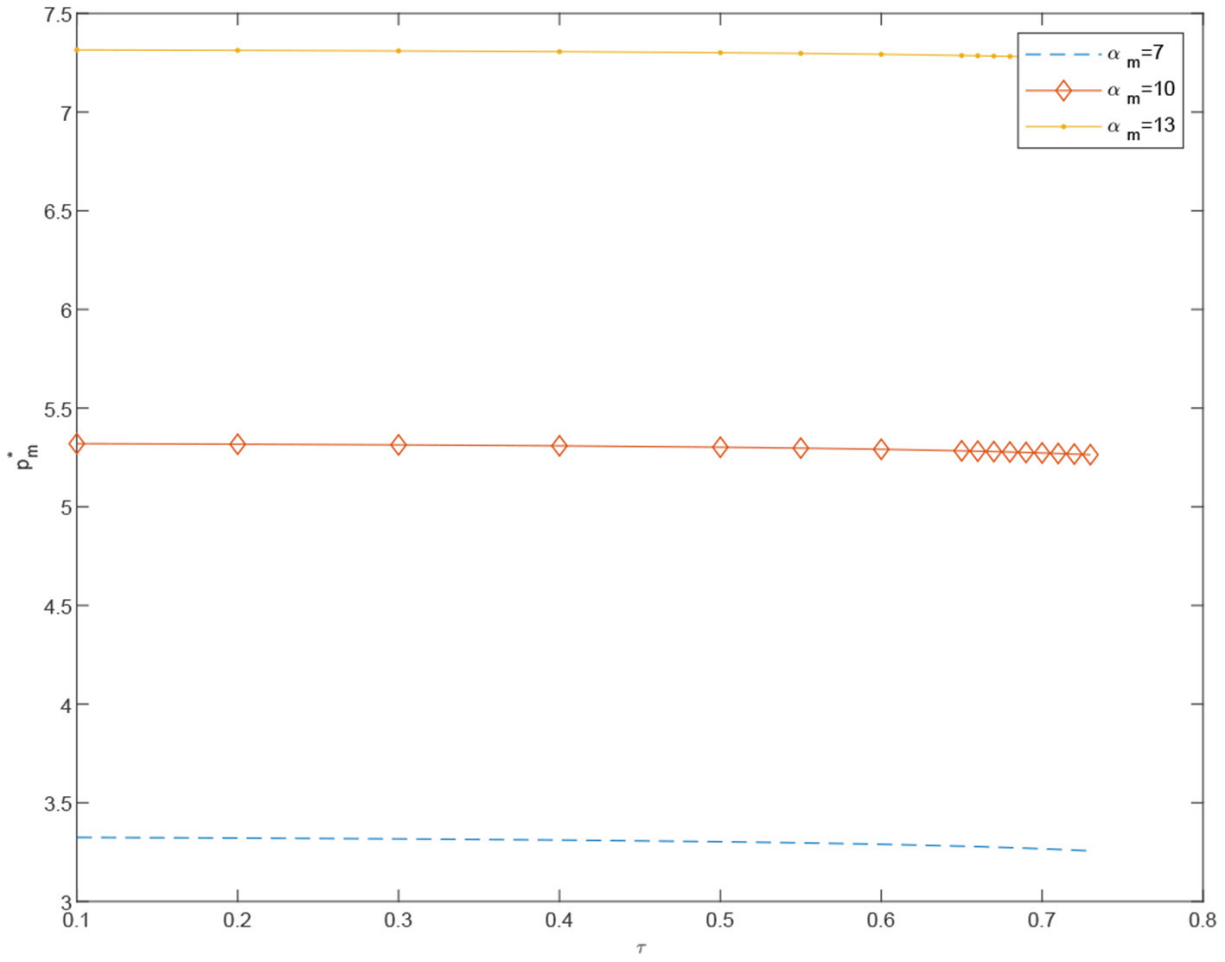
The effect of τ on $p_{m-O}^{*}$ under different α_*m*_.

**Figure 13:**
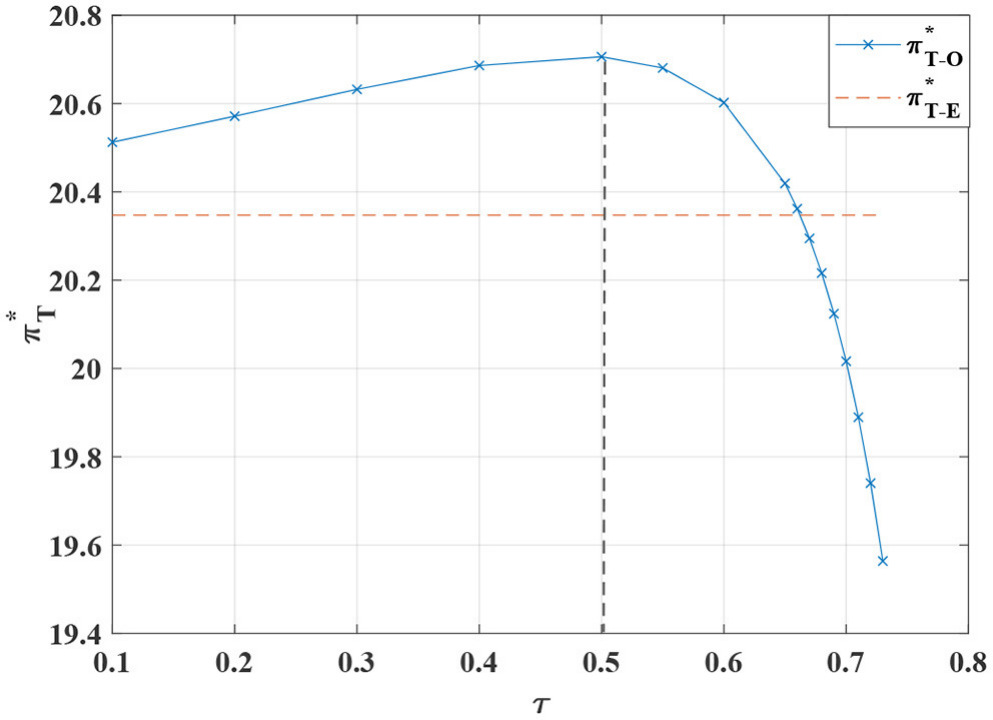
The impact of τ on the overall optimal profit of the system.

**Figure 14:**
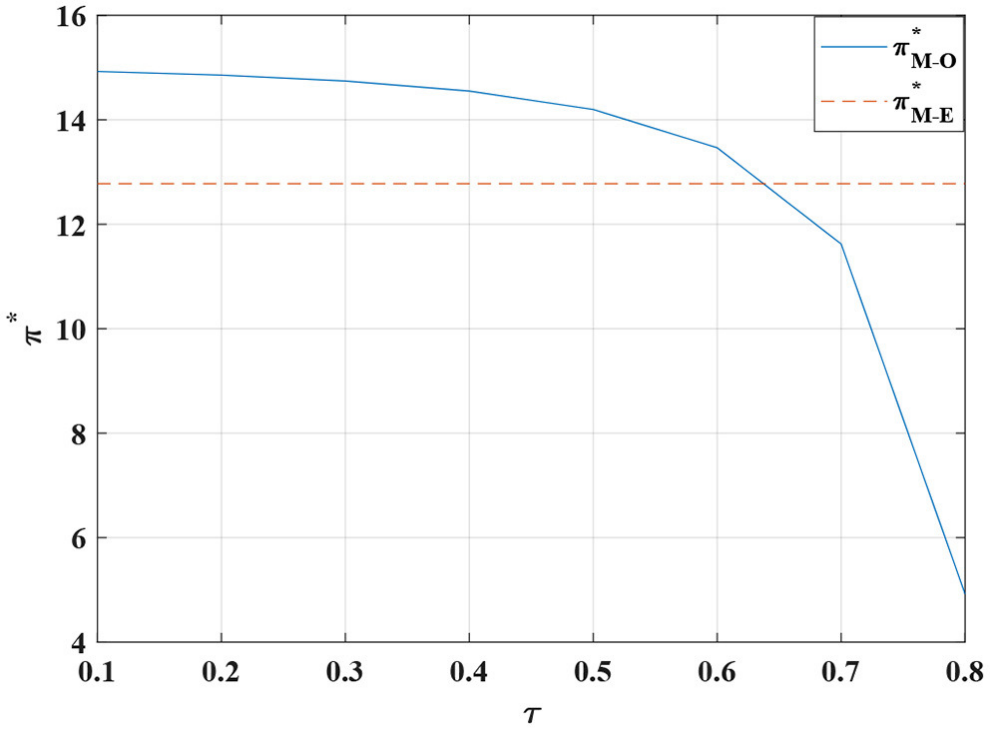
The impact of τ on the manufacturer's optimal profit.

**Figure 15:**
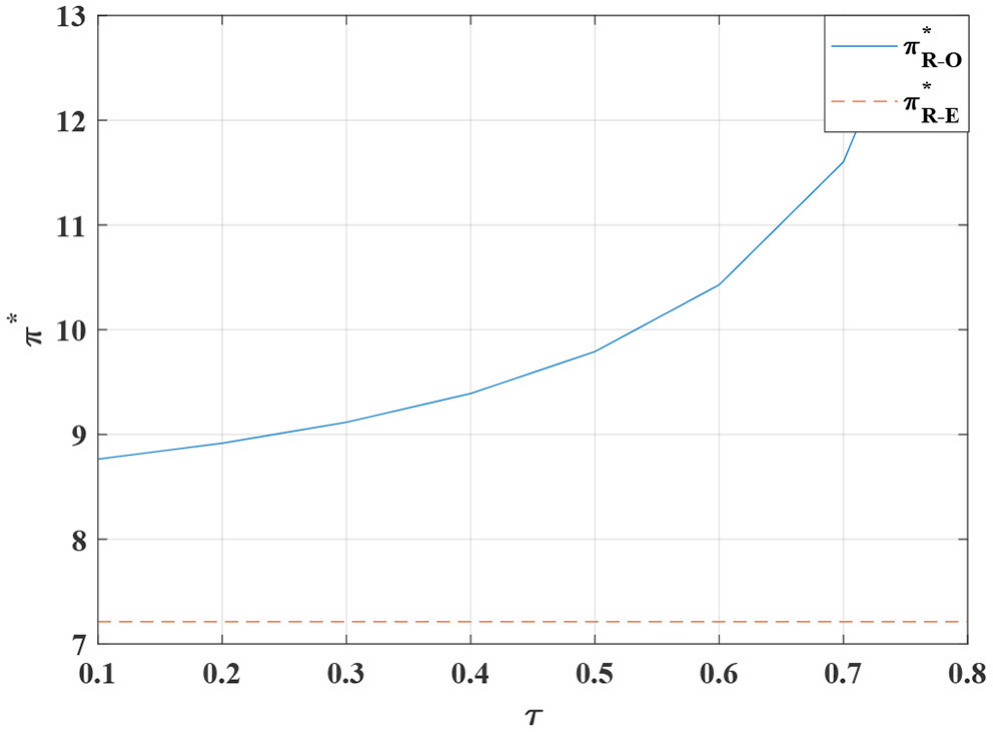
The influence of τ on the retailer's optimal profit.

In Figures 6–15, as α_*m*_ increases, the price of standardized products *p*_*e*_ decreases, but the price of customized products *p*_*m*_ increases significantly. In the cost-sharing contract, the change of τ will not significantly cause the change of *p*_*m*_, but *p*_*e*_ increases with an increase in τ. We conclude that in a cost-sharing contract, the manufacturer takes the initiative to bear part of the cost of standardized channels. The retailer can reduce part of the cost of standardized channel construction, so as to improve their enthusiasm for channel construction, and further promote the perfection of standardized channels. Consumers can enjoy a better service experience with standardized products. Therefore, the price of standardized products will be partially increased.

**Proposition 10**. We observe that when the value of τ is between 0.1 and 0.55, the total profit of the system is constantly increasing in Figure 13. When the value of τ is higher than 0.55, the total profit of the system declines exponentially. The proportion of the manufacturer's burden of the retailer's standardized costs is not linearly related. Moreover, when the manufacturer covers 50% of the standardized costs, the total profit of the system is maximized. When the manufacturer shoulders more than 55% of the cost of standardized channels, the conflict is not mitigated. When the manufacturer absorbs more than 55% of such costs, the total profit falls sharply. As a result, the manufacturer should induce the retailer to strive to sell standardized products by setting a reasonable cost-sharing ratio.

The cost-sharing coordination model has an improved effect on the overall profitability of the channel, with the manufacturer bearing part of the standardized costs for the retailer, increasing the retailer's incentive to participate in customization. In addition, the price of standardized and customized products are relatively stable. Customers obtain higher consumer surplus, stimulating the demand for channel standardization and customization and further driving manufacturers to produce more customized and standardized products.

**Figure 16:**
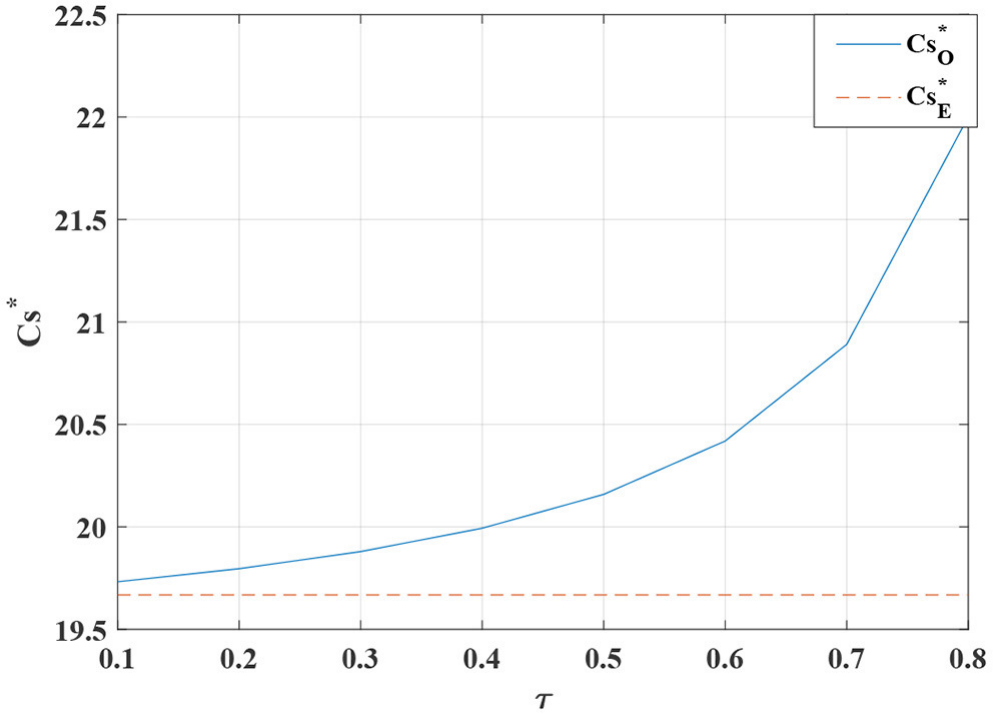
The influence of τ on the optimal consumer surplus.

In Figures 13–16. It is observed that when the proportion of standardized costs shared by the manufacturer is within a reasonable constraint, the cost-sharing contract can not only effectively motivate the retailer to improve the level of channel sales service, but also achieve Pareto improvement in the interests of the manufacturer, the retailer and the consumers. It will better promote the coordination of the entire system. The reason is that when the manufacturer bears part of the standardized channel construction cost for the retailer, this cost-sharing behavior will have an incentive effect on the retailer, prompting it to actively operate the standardized channel. Therefore, more diversified standardized products and more complete product services provide consumers with a better product consumption experience. Thereby enhancing consumers' willingness to purchase standardized products, expanding market demand, and improving retail sales. For the manufacturer, the cost-sharing contract brings more profit than the cost itself, so it can also obtain a higher level of profit.

Compared with the decentralized dual channel case, the cost-sharing contract has better overall supply chain performance and sales price in both channels. By sharing the cost of the standardized channel, the manufacturer alleviates the channel conflict and retailers' incentive is improved. Although the sales price of customized and standardized products are both slightly higher than those before the channel coordination, but consumers have access to a higher level of customization to meet their diverse consumer needs. With the increase of consumers' income and consumption level, high-quality customized products will appeal to a constantly increasing number of consumers.

## 5. Conclusions

In this paper, we develop a dual channel two-level supply chain (manufacturer-retailer) system where standardized products are sold through the retail channel and customized products are sold online by the manufacturer. The results show the demand for customized and standardized products and the profits of the dual channel are affected by the price, the degree of customization and the degree of standardization of the products themselves. Sensitivity analysis is also performed to examine the effect of the fluctuations of various input parameters. The results show that changing input parameters λ_*m*_ and τ has a significant impact on the optimal decisions of the supply chain system. We build three different scenarios by Stackelberg games, a centralized dual channel decision, a decentralized dual channel decision, and a cost-sharing dual channel decision. Under each scenario, we obtain the optimal degree of customization level, the optimal degree of standardization level, the optimal price, and the optimal profit, and we draw the following conclusions.

1. Under the centralized scenario, the system can achieve the best overall system profitability. However, under the decentralized scenario, the customized and standardized dual-channel system does not achieve the coordinated state of the dual-channel centralized system. With the reasonable cost sharing ratio, the cost sharing contract can effectively alleviate and balances the profits of channel members under the decentralized scenario.
2. The increase in the degree of customization can improve the overall profit of the system and the satisfaction of consumers. In addition, customization strategies can help manufacturers close the competitive gap between direct customization channels and retailers' traditional standardized channels in a decentralized system. On the other hand, managers need to balance the benefits of increased customization with the loss of large costs.
3. The demand in the customized market is positively related to the price of customized products and the degree of customization. However, considering the degree of market competition, the degree of customization under decentralized scenario is greater than that under centralized scenario. In addition, there is no linear growth relationship between the profit of the system and the demand of the customized market. Therefore, managers need to fulfill the market demand as much as possible, so as not only to meet the demand of the consumers, but also to actively attract new consumers. On the other hand, customized decisions need to be further judged based on market conditions.
4. The reasons for the conflict between customized channels and standardized channels are complex. Except incompatible goals, they also need to consider the customized channel market share, the channel substitutability, and the impact of customization sensitivity and standardization sensitivity on those channels.

To summarize this study, we can see that the manufacturer should increase the research and development investment of customization. It can helps to establish the product mode of diversification and customization through a comprehensive transformation from its original business and management processes to meet the individual needs of the customers. This transformation leads to a rapid response and a rapid delivery of the customized orders. The potential market for customized products will then inevitably increase. It suggests that the manufacturer could also share the cost of standardization with the retailer in case to motivate the retailers to participate in the customization process.

## Data availability statement

The original contributions presented in the study are included in the article/supplementary material, further inquiries can be directed to the corresponding author/s.

## Author contributions

BD: conceptualization and methodology. JZ: investigation. ZL and BD: writing—original draft preparation. BD, WS, and YJ: writing—review and editing. YJ: funding acquisition. All authors have read and approved the final manuscript.

## Funding

This work is supported by National Natural Science Foundation of China (71771128, 71402075, 72172069, and 71502088), Fundamental Research Funds for the Provincial Universities of Zhejiang (SJWZ2021002 and SJWY2021001), K.C. Wong Magna Fund in Ningbo University.

## Conflict of Interest

The authors declare that the research was conducted in the absence of any commercial or financial relationships that could be construed as a potential conflict of interest.

## Publisher's Note

All claims expressed in this article are solely those of the authors and do not necessarily represent those of their affiliated organizations, or those of the publisher, the editors and the reviewers. Any product that may be evaluated in this article, or claim that may be made by its manufacturer, is not guaranteed or endorsed by the publisher.

## Appendix

### Proofs for theorems

*Proof for Theorem* *1**:* The profit function of f the centralized dual channel supply chain is

(A1)
$$\begin{array}{l} \Pi_{T}\left(p_{e},p_{m},e,m\right)=\left(p_{e}-c_{e}\right)D_{e}+\left(p_{m}-c_{m}\right)D_{m}-\frac{K_{m}}{2}m^{2}-\frac{K_{e}}{2}e^{2} \end{array}$$

Then the Hessian matrix is,

(A2)
$$\begin{array}{l} H=\left[\begin{array}{cccc} -k_{e} & 0 & \frac{-\theta \lambda_{e}}{1-\theta^{2}} & \frac{\lambda_{e}}{1-\theta^{2}}\\ 0 & -k_{m} & \frac{\lambda_{m}}{1-\theta^{2}} & \frac{-\theta \lambda_{m}}{1-\theta^{2}}\\ \frac{-\theta \lambda_{e}}{1-\theta^{2}} & \frac{\lambda_{m}}{1-\theta^{2}} & \frac{-2}{1-\theta^{2}} & \frac{2\theta }{1-\theta^{2}}\\ \frac{\lambda_{e}}{1-\theta^{2}} & \frac{-\theta \lambda_{m}}{1-\theta^{2}} & \frac{2\theta }{1-\theta^{2}} & \frac{-\text{2}}{1-\theta^{2}} \end{array}\right] \end{array}$$

To ensure the existence of the optimal solution, *H* must be negative definite. We thus have $4k_{e}k_{m}\left(1-\theta^{2}\right)-2k_{e}{\lambda_{m}}^{2}-2k_{m}{\lambda_{e}}^{2}+{\lambda_{e}}^{2}{\lambda_{m}}^{2}>0$. We assume the first order condition equations above are 0, then we get

(A3)
$$\begin{array}{l} \{\begin{array}{l} \frac{\partial \pi_{T}}{\partial e}=\text{(}p_{e}-c_{e}\text{)}\frac{\lambda_{e}}{1-\theta^{2}}+\left(p_{m}-{\text{c}}_{m}\right)\frac{-\theta \lambda_{e}}{1-\theta^{2}}-k_{e}e\text{= 0}\\ \frac{\partial \pi_{T}}{\partial m}=\text{(}p_{e}-c_{e}\text{)}\frac{-\theta \lambda_{m}}{1-\theta^{2}}+\left(p_{m}-c_{m}\right)\frac{\lambda_{m}}{1-\theta^{2}}-k_{m}m\text{= 0}\\ \frac{\partial \pi_{T}}{\partial p_{e}}=\frac{\alpha_{e}-\theta \alpha_{m}-p_{e}+\theta p_{m}+\lambda_{e}e-\theta \lambda_{m}m}{1-\theta^{2}}+\frac{-1}{1-\theta^{2}}\text{(}p_{e}-c_{e}\text{)}\\ \;+\left(p_{m}-c_{m}\right)\frac{\theta }{1-\theta^{2}}=0\\ \frac{\partial \pi_{T}}{\partial p_{m}}=\text{(}p_{e}-c_{e}\text{)}\frac{\theta }{1-\theta^{2}}+\frac{\alpha_{m}-\theta \alpha_{e}-p_{m}+\theta p_{e}+\lambda_{m}m-\theta \lambda_{e}e}{1-\theta^{2}}\\ \;+\left(p_{m}-c_{m}\right)\frac{-1}{1-\theta^{2}}=0 \end{array} \end{array}$$

we solve the first order partial derivatives with respect to *p*_*e*_, *p*_*m*_, *e* and *m* respectively, and then we can get the optimal results simultaneously.

(A4)
$$\begin{array}{l} e_{C}^{*}=\frac{-\theta \lambda_{e}\left(2k_{m}\right)\left(\alpha_{m}-c_{m}\right)+\lambda_{e}\left(2k_{m}-\lambda_{m}^{2}\right)\left(\alpha_{e}-c_{e}\right)}{4k_{m}k_{e}\left(1-\theta^{2}\right)-2k_{e}\lambda_{m}^{2}-2k_{m}\lambda_{e}^{2}+\lambda_{e}^{2}\lambda_{m}^{2}} \end{array}$$

(A5)
$$\begin{array}{l} m_{C}^{*}=\frac{-\theta \lambda_{m}\left(2k_{e}\right)\left(\alpha_{e}-c_{e}\right)+\lambda_{m}\left(2k_{e}-\lambda_{e}^{2}\right)\left(\alpha_{m}-c_{m}\right)}{4k_{m}k_{e}\left(1-\theta^{2}\right)-2k_{e}\lambda_{m}^{2}-2k_{m}\lambda_{e}^{2}+\lambda_{e}^{2}\lambda_{m}^{2}} \end{array}$$

(A6)
$$\begin{array}{l} p_{e-C}^{*}=\frac{-\theta \lambda_{e}^{2}\left(2k_{m}\right)\left(\alpha_{m}-c_{m}\right)+\left(4k_{m}k_{e}\left(1-\theta^{2}\right)-2k_{e}\lambda_{m}^{2}\right)\alpha_{e}}{2}\\ \;+\frac{\left(4k_{m}k_{e}\left(1-\theta^{2}\right)-2k_{e}\lambda_{m}^{2}-4k_{m}\lambda_{e}^{2}+2\lambda_{e}^{2}\lambda_{m}^{2}\right)c_{e}}{2} \end{array}$$

(A7)
$$\begin{array}{l} p_{m-C}^{*}=\frac{-\theta \lambda_{m}^{2}\left(2k_{e}\right)\left(\alpha_{e}-c_{e}\right)+\left(4k_{m}k_{e}\left(1-\theta^{2}\right)-2k_{m}\lambda_{e}^{2}\right)\alpha_{m}}{2}\\ \;+\frac{\left(4k_{m}k_{e}\left(1-\theta^{2}\right)-4k_{e}\lambda_{m}^{2}-2k_{m}\lambda_{e}^{2}+2\lambda_{e}^{2}\lambda_{m}^{2}\right)c_{m}}{2} \end{array}$$

The optimal profit under the centralized case is

(A8)
$$\begin{array}{l} \pi_{T-C}^{*}=\left(p_{e-C}^{*}-c_{e}\right)\frac{\alpha_{e}-\theta \alpha_{m}-p_{e-C}^{*}+\theta p_{m-C}^{*}+\lambda_{e}e_{C}^{*}-\theta \lambda_{m}m_{C}^{*}}{1-\theta^{2}}\\ \;+\left(p_{m-C}^{*}-c_{m}\right)\frac{\alpha_{m}-\theta \alpha_{e}-p_{m-C}^{*}+\theta p_{e-C}^{*}+\lambda_{m}m_{C}^{*}-\theta \lambda_{e}e_{C}^{*}}{1-\theta^{2}}\\ \;-\frac{k_{m}{m_{C}^{*}}^{2}}{2}-\frac{k_{e}{e_{C}^{*}}^{2}}{2} \end{array}$$

*Proof for Theorem* *2**:* The profit function of the retailer is,

(A9)
$$\begin{array}{l} \pi_{\text{R}-E}=\left(p_{e}-\omega \right)D_{e}-\frac{k_{e}e^{2}}{2} \end{array}$$

The first order condition (FOC),

(A10)
$$\begin{array}{l} D_{e}=\frac{\alpha_{e}-\theta \alpha_{m}-p_{e}+\theta p_{m}+\lambda_{e}e-\theta \lambda_{m}m}{1-\theta^{2}} \end{array}$$

Henceforth the first order condition of π_R_ on *p*_*e*_ and *e* are

(A11)
$$\begin{array}{l} \{\begin{array}{l} \frac{\partial \pi_{\text{R-E}}}{de}=\frac{\lambda_{e}\left(p_{e}-\omega \right)}{1-\theta^{2}}-k_{e}e=0\\ \frac{\partial \pi_{\text{R-E}}}{dp_{e}}=D_{e}+\left(p_{e}-\omega \right)\frac{-1}{1-\theta^{2}}\\ \;=\frac{\alpha_{e}-\theta \alpha_{m}-p_{e}+\theta p_{m}+\lambda_{e}e-\theta \lambda_{m}m}{1-\theta^{2}}+\frac{-\left(p_{e}-\omega \right)}{1-\theta^{2}}=0 \end{array} \end{array}$$

Then the Hessian matrix is

(A12)
$$\begin{array}{l} G=\left[\begin{array}{c} -k_{e}\frac{\lambda_{e}}{1-\theta^{2}}\\ \frac{\lambda_{e}}{1-\theta^{2}}\frac{\text{- 2}}{1-\theta^{2}} \end{array}\right] \end{array}$$

*G* must be negative definite. Then we assume that $\text{2}\left(1-\theta^{2}\right)k_{e}-{\lambda_{e}}^{\text{2}}>0$. Thus the retailer's profit function is jointly strictly concave in *p*_*e*_ and *e*.

We assume the first order condition equations above are 0, then we get

(A13)
$$\begin{array}{l} \{\begin{array}{l} {\text{p}}_{\text{e}}\left(p_{m},m\right)=\frac{\alpha_{e}-\theta \alpha_{m}+\theta p_{m}-M\lambda_{e}\omega -\theta \lambda_{m}m+\omega }{2-M\lambda_{e}}\\ e\left(p_{m},m\right)=M\left(\frac{\alpha_{e}-\theta \alpha_{m}+\theta p_{m}-M\lambda_{e}\omega -\theta \lambda_{m}m+\omega }{2-M\lambda_{e}}-\omega \right) \end{array} \end{array}$$

Solving for the manufacturer's profit function.

(A14)
$$\begin{array}{l} \pi_{\text{M-E}}=\left(\omega -c_{e}\right)D_{e}+\left(p_{m}-c_{m}\right)D_{m}-\frac{k_{m}m^{2}}{2} \end{array}$$

The first order condition

(A15)
$$\begin{array}{l} \{\begin{array}{l} {\text{p}}_{\text{e}}\left(p_{m},m\right)=\frac{\alpha_{e}-\theta \alpha_{m}+\theta p_{m}-M\lambda_{e}\omega -\theta \lambda_{m}m+\omega }{2-M\lambda_{e}}\\ e\left(p_{m},m\right)=M\left(\frac{\alpha_{e}-\theta \alpha_{m}+\theta p_{m}-M\lambda_{e}\omega -\theta \lambda_{m}m+\omega }{2-M\lambda_{e}}-\omega \right) \end{array} \end{array}$$

Henceforth, we can get the demand function

(A16)
$$\begin{array}{l} \{\begin{array}{c} D_{e}=\frac{\alpha_{e}-\theta \alpha_{m}-\frac{\alpha_{e}-\theta \alpha_{m}+\theta p_{m}-M\lambda_{e}\omega -\theta \lambda_{m}m+\omega }{2-M\lambda_{e}}+\theta p_{m}+\lambda_{e}M\left(\frac{\alpha_{e}-\theta \alpha_{m}+\theta p_{m}-M\lambda_{e}\omega -\theta \lambda_{m}m+\omega }{2-M\lambda_{e}}-\omega \right)-\theta \lambda_{m}m}{1-\theta^{2}}\\ D_{m}=\frac{\alpha_{m}-\theta \alpha_{e}-p_{m}+\theta \frac{\alpha_{e}-\theta \alpha_{m}+\theta p_{m}-M\lambda_{e}\omega -\theta \lambda_{m}m+\omega }{2-M\lambda_{e}}+\lambda_{m}m-\theta \lambda_{e}M\left(\frac{\alpha_{e}-\theta \alpha_{m}+\theta p_{m}-M\lambda_{e}\omega -\theta \lambda_{m}m+\omega }{2-M\lambda_{e}}-\omega \right)}{1-\theta^{2}} \end{array} \end{array}$$

so the Hessian matrix is

(A17)
$$\begin{array}{l} W=\left[\begin{array}{cc} -k_{m} & \frac{Q\lambda_{m}}{\left(1-\theta^{2}\right)\left(2-M\lambda_{e}\right)}\\ \frac{Q\lambda_{m}}{\left(1-\theta^{2}\right)\left(2-M\lambda_{e}\right)} & \frac{-2Q}{\left(1-\theta^{2}\right)\left(2-M\lambda_{e}\right)} \end{array}\right] \end{array}$$

In order to ensure the existence of the optimal solution, *W* must be negative definite. We assume that $\frac{2Qk_{m}\left(\left(1-\theta^{2}\right)\left(2-M\lambda_{e}\right)\right)-{\left(Q\lambda_{m}\right)}^{2}}{{\left(1-\theta^{2}\right)}^{2}{\left(2-M\lambda_{e}\right)}^{2}}>0$, which implies $2Qk_{m}\left(\left(1-\theta^{2}\right)\left(2-M\lambda_{e}\right)\right)>{\left(Q\lambda_{m}\right)}^{2}$ and *Mλ*_*e*_ < 2.

The optimal price and the degree of customization are obtained as

(A18)
$$\begin{array}{l} \{\begin{array}{l} p_{m-E}^{*}=\frac{\alpha_{m}}{2}+\frac{\lambda_{m}\left[\left(\omega -c_{e}\right)\frac{-2\theta \lambda_{m}}{2Nk_{m}-Q\lambda_{m}^{2}}+\left(\alpha_{m}+\frac{-\theta \alpha_{e}+\left(\theta +\theta M\lambda_{e}\right)\omega -\theta c_{e}}{Q}-c_{\text{m}}\right)\frac{Q\lambda_{m}}{2Nk_{m}-Q\lambda_{m}^{2}}\right]}{2}\\ \;+\frac{-\theta \alpha_{e}+\left(\theta +\theta M\lambda_{e}\right)\omega -\theta c_{e}}{2Q}+\frac{c_{\text{m}}}{2}\\ m_{E}^{*}\;=\left(\omega -c_{e}\right)\frac{-2\theta \lambda_{m}}{2Nk_{m}-Q\lambda_{m}^{2}}+\left(\alpha_{m}+\frac{-\theta \alpha_{e}+\left(\theta +\theta M\lambda_{e}\right)\omega -\theta c_{e}}{Q}-c_{\text{m}}\right)\frac{Q\lambda_{m}}{2Nk_{m}-Q\lambda_{m}^{2}} \end{array} \end{array}$$

Then we can calculate the optimal standardized product price and standardization degree

(A19)
$$\begin{array}{l} \{\begin{array}{ll} p_{e-E}^{*} & =\frac{\alpha_{e}-\theta \alpha_{m}+\theta p_{m-E}^{*}-M\lambda_{e}\omega -\theta \lambda_{m}m_{E}^{*}+\omega }{2-M\lambda_{e}}\\ e_{E}^{*} & =M\left(\frac{\alpha_{e}-\theta \alpha_{m}+\theta p_{m-E}^{*}-\theta \lambda_{m}m_{E}^{*}-\omega }{2-M\lambda_{e}}\right) \end{array} \end{array}$$

From the above equilibrium values we derive the retailer' profit, and the manufacturer's profit.

*Proof for Theorem* *3**:* In the cost-sharing contract, we solve for retailer's profit function first.

(A20)
$$\begin{array}{l} \pi_{\text{R-O}}=\left(p_{e}-\omega \right)D_{e}-\left(1-\tau \right)\frac{k_{e}e^{2}}{2} \end{array}$$

Then the Hessian matrix is

(A21)
$$\begin{array}{l} Z=\left[\begin{array}{cc} -\left(1-\tau \right)k_{e} & \frac{\lambda_{e}}{1-\theta^{2}}\\ \frac{\lambda_{e}}{1-\theta^{2}} & \frac{\text{- 2}}{1-\theta^{2}} \end{array}\right] \end{array}$$

In order to ensure that the optimal value exists, *Z* must be negative definite. Then we assume that $\text{2}\left(1-\tau \right)\left(1-\theta^{2}\right)k_{e}-{\lambda_{e}}^{\text{2}}>0$. Thus the retailer's profit function is jointly strictly concave in *p*_*e*_ and *e*. We assume the first order condition equation above is 0, then we get

(A22)
$$\begin{array}{l} \{\begin{array}{l} \text{p\_e}\left(p_{m},m\right)=\frac{\alpha_{e}-\theta \alpha_{m}+\theta p_{m}-A\lambda_{e}\omega -\theta \lambda_{m}m+\omega }{2-A\lambda_{e}}\\ e\left(p_{m},m\right)=A\left(p_{e}-\omega \right) \end{array} \end{array}$$

Solving for the manufacturer's profit function.

(A23)
$$\begin{array}{l} \pi_{M-O}=\left(\omega -C_{e}\right)D_{e}+\left(p_{m}-C_{m}\right)D_{m}-\frac{k_{m}m^{2}}{2}-\frac{\tau k_{e}e^{2}}{2} \end{array}$$

The first order condition of π_*M*−*O*_ on *m* is

(A24)
$$\begin{array}{l} \frac{\partial \pi_{\text{M}-O}}{\partial m}=\left(\omega -c_{e}\right)D_{e}^{\prime }+\left(p_{m}-c_{m}\right)D_{m}^{\prime }-k_{m}m=0 \end{array}$$

The first order condition of π_*M*−*O*_ on *p*_*m*_ is

(A25)
$$\begin{array}{l} \frac{\partial \pi_{\text{M}-O}}{\partial_{\text{m}}}=D_{e}^{\prime }\left(\omega -c_{e}\right)+D_{\text{m}}+\left(p_{m}-c_{\text{m}}\right)D_{\text{m}}^{\prime }=0 \end{array}$$

so the Hessian matrix is

(A26)
$$\begin{array}{l} Y=\left[\begin{array}{cc} -k_{m} & \frac{F\lambda_{m}}{\left(1-\theta^{2}\right)\left(2-A\lambda_{e}\right)}\\ \frac{F\lambda_{m}}{\left(1-\theta^{2}\right)\left(2-A\lambda_{e}\right)} & \frac{-2F}{\left(1-\theta^{2}\right)\left(2-A\lambda_{e}\right)} \end{array}\right] \end{array}$$

we assume that $\text{2}\left(1-\tau \right)\left(1-\theta^{2}\right)k_{e}-{\lambda_{e}}^{\text{2}}>0$. Thus the manufacturer's profit function is strictly concave in *p*_*m*_ and *m*. We assume the first order condition equations above are 0, then we get

The optimal the degree of customization under the cost-sharing contract is

(A27)
$$\begin{array}{l} m_{O}^{*}=\left(\omega -c_{e}\right)\frac{-2\theta \lambda_{m}}{2Ek_{m}-F\lambda_{m}^{2}}\\ \;+\left(\alpha_{m}+\frac{-\theta \alpha_{e}+\left(\theta +\theta A\lambda_{e}\right)\omega -\theta c_{e}}{F}-c_{\text{m}}\right)\frac{F\lambda_{m}}{2Ek_{m}-F\lambda_{m}^{2}} \end{array}$$

The optimal price of the customized products under the cost-sharing contract is

(A28)
$$\begin{array}{l} p_{m-O}^{*}=\frac{\alpha_{m}}{2}\\ +\frac{\lambda_{m}\left[\left(\omega -c_{e}\right)\frac{-2\theta \lambda_{m}}{2Ek_{m}-F\lambda_{m}^{2}}+\left(\alpha_{m}+\frac{-\theta \alpha_{e}+\left(\theta +\theta A\lambda_{e}\right)\omega -\theta c_{e}}{F}-c_{\text{m}}\right)\frac{F\lambda_{m}}{2Ek_{m}-F\lambda_{m}^{2}}\right]}{2}\\ +\frac{-\theta \alpha_{e}+\left(\theta +\theta A\lambda_{e}\right)\omega -\theta c_{e}}{2F}+\frac{c_{\text{m}}}{2} \end{array}$$

Then we can get the optimal values for *p*_*e*_ and *e*

(A29)
$$\begin{array}{l} e_{O}^{*}=\frac{\alpha_{e}-\theta \alpha_{m}+\theta p_{m-O}^{*}-\theta \lambda_{m}m_{O}^{*}-\omega }{2-A\lambda_{e}} \end{array}$$

(A30)
$$\begin{array}{l} p_{e-O}^{*}=\frac{\alpha_{e}-\theta \alpha_{m}+\theta p_{m-O}^{*}-A\lambda_{e}\omega -\theta \lambda_{m}m_{O}^{*}+\omega }{2-A\lambda_{e}} \end{array}$$

From the above equilibrium values we derive the retailer' profit, and the manufacturer's profit.
